# Genetic encoding and expression of RNA origami cytoskeletons in synthetic cells

**DOI:** 10.1038/s41565-025-01879-3

**Published:** 2025-03-17

**Authors:** Mai P. Tran, Taniya Chakraborty, Erik Poppleton, Luca Monari, Maja Illig, Franziska Giessler, Kerstin Göpfrich

**Affiliations:** 1 Biophysical Engineering Group, Heidelberg University, Center for Molecular Biology of Heidelberg University (ZMBH), Heidelberg, Germany; 2 Biophysical Engineering Group, Max Planck Institute for Medical Research, Heidelberg, Germany; 3 Biomolecular Mechanics Group, Max Planck Institute for Polymer Research, Mainz, Germany

**Keywords:** RNA nanotechnology, Biomaterials

## Abstract

Bottom-up synthetic biology seeks to engineer a cell from molecular building blocks. Using DNA nanotechnology, building blocks, such as cytoskeletons, have been reverse-engineered. However, DNA nanostructures rely on chemical synthesis and thermal annealing, and therefore synthetic cells cannot produce them from their constituents such as nucleotides. Here we introduce RNA origami cytoskeleton mimics as alternative nucleic acid-based molecular hardware for synthetic cells, which we express directly inside giant unilamellar lipid vesicles (GUVs) containing a DNA template and a polymerase, chemically fuelled by feeding nucleotides from the outside. We designed RNA origami tiles that fold upon transcription and self-assemble into micrometre-long, three-dimensional RNA origami nanotubes under isothermal conditions. We observe that sequence mutations on the DNA template lead to RNA origami nanotubes and closed-ring phenotypes. Molecular dynamics simulations show that these phenotypic transitions are governed by alterations in the stability of RNA secondary structures. In addition, we achieve cortex formation with aptamer-functionalized RNA nanotubes and show that nanotube polymerization leads to membrane deformation. Altogether, our data suggest that the expression of RNA origami-based hardware will help to explore active, evolvable and RNA-based synthetic cells.

## Abstract (web summary)

This study introduces RNA origami nanotubes as self-assembling cytoskeleton mimics for synthetic cells. Expressed in vesicles from DNA templates, these RNA structures reach micrometre lengths, deform membranes and exhibit different phenotypes.

## Main

**Fig. 1:**
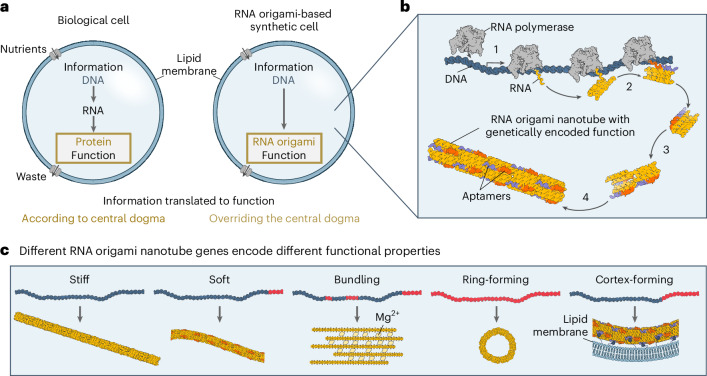
Motivation and conceptualization for engineering RNA origami-based hardware for synthetic cells. **a**, Biological cells function according to the central dogma (DNA serves as a template for RNA, and RNA directs protein synthesis), necessitating the involvement of about 150 genes in the translational process. By contrast, a synthetic cell built with RNA origami requires fewer genes while maintaining evolvability. **b**, Mechanism of co-transcriptional RNA origami. A DNA template is transcribed by RNA polymerase, whereby the RNA folds up into tiles that self-assemble into higher-order RNA origami nanotubes. The folding steps are numbered: the nascent RNA folds first into local secondary structures (stem-loops), followed by the formation of long-distance tertiary structures through internal kissing loops and finally oligomerization via various quaternary interactions (external kissing loops, overhangs and aptamers). **c**, Information–function correlation. Mutations on the DNA template result in RNA origami nanotubes with different properties.

Bottom-up synthetic biology has the visionary goal of engineering a cell—the minimal unit of life—from non-living molecular components^1,2^. Central to this effort is the imperative for synthetic cells to construct their own hardware using an intrinsic genome and environmental nutrients, which would support evolutionary adaptation through natural selection. The most obvious approach is to leverage the information–function relationship that biology already developed, namely, the ‘central dogma of molecular biology’, which states that information flows from DNA (information) to RNA (amplification and regulation) and finally to protein (function)^3^ (Fig. 1a). Entrenched in nature’s evolutionary processes, this pathway remains the only known fully functional chassis for life^4^. The central dogma, however, is a relatively complicated minimal system.

Protein production in GUVs, although possible^5–7^, presents challenges such as low encapsulation efficiency, limited post-translation modification, cross-talk and limited lifespan^8^. Moreover, a synthetic cell functioning according to the central dogma would require over 150 genes for the transcription–translation machinery alone^4,9,10^. At the onset of life, simpler processes must have sustained self-replication and evolution^11^. One potential solution is to ‘short-circuit’ the central dogma and create a system with only a single step between genotype and phenotype. DNA nanotechnology^12^, fuelled by the development of DNA origami^13^, has facilitated the creation of structural and, to some extent, functional analogues of cellular proteins, which have been reconstituted in GUVs^14–16^. These represent initial steps towards a protein-free machinery for synthetic cells^17,18^.

Despite notable successes, there are conceptual challenges related to the use of DNA nanotechnology for the construction of synthetic cells. (i) Using DNA as both genetic and functional material simultaneously introduces significant design constraints. Separating the nucleic acids into a linear, double-stranded information payload and a single-stranded functional unit increases freedom in the design space. (ii) Functionality typically requires chemical functionalization of the DNA, for example, cholesterol for membrane anchoring^19^. This further complicates the encoding and production of these structures within a vesicle. (iii) Vesicles containing DNA nanostructures typically operate in equilibrium, which is not consistent with sustaining life. (iv) Finally, the implementation of evolution is not straightforward. Although it may be possible to genetically encode DNA origami in vesicles^20^, purification and annealing steps were still required, which is not consistent with an autonomous mutation and selection cycle.

We propose here that these challenges could be addressed using co-transcriptional RNA origami for the construction of synthetic cellular hardware. RNA origami can be genetically encoded in synthetic DNA templates^21–23^ using a fraction of the existing machinery from the central dogma—only a single protein, RNA polymerase. An RNA origami-based synthetic cell would thus, at least initially, require an environment where a single protein is abundant. Furthermore, the RNA origami folding mechanism^21,24^ resembles that of proteins—a single polymer chain transcribed by a polymerase undergoes a series of co-transcriptional folding steps (Fig. 1b). Structures can be rationally designed, allowing for the creation of a variety of RNA origami structures while offering greater versatility than DNA.

In this paper, we produce RNA origami cytoskeletons from a DNA template as their ‘genome’ inside of GUVs under the consumption of chemical fuel in the form of rNTPs (ATP, GTP, UTP and CTP) (Fig. 1b). Small variations in the DNA template sequence significantly impact the structure and function of RNA origami nanotubes (Fig. 1c).

## Results

### Expression of RNA origami within GUVs

We first aimed to encapsulate the DNA template and the transcription machinery required for the production of RNA origami inside GUVs. Compared with in vitro transcription in bulk, we first have to ensure that transcription is indeed happening inside the compartment and not before encapsulation. Therefore, an external trigger is needed. Second, owing to the limited reaction volume of the GUV, nutrient depletion and waste accumulation would stall and favour abortive initiation of the transcription process^25^. We thus introduce two distinct control schemes: restriction of the essential cofactor, Mg^2+^, and restriction of rNTPs.

**Fig. 2:**
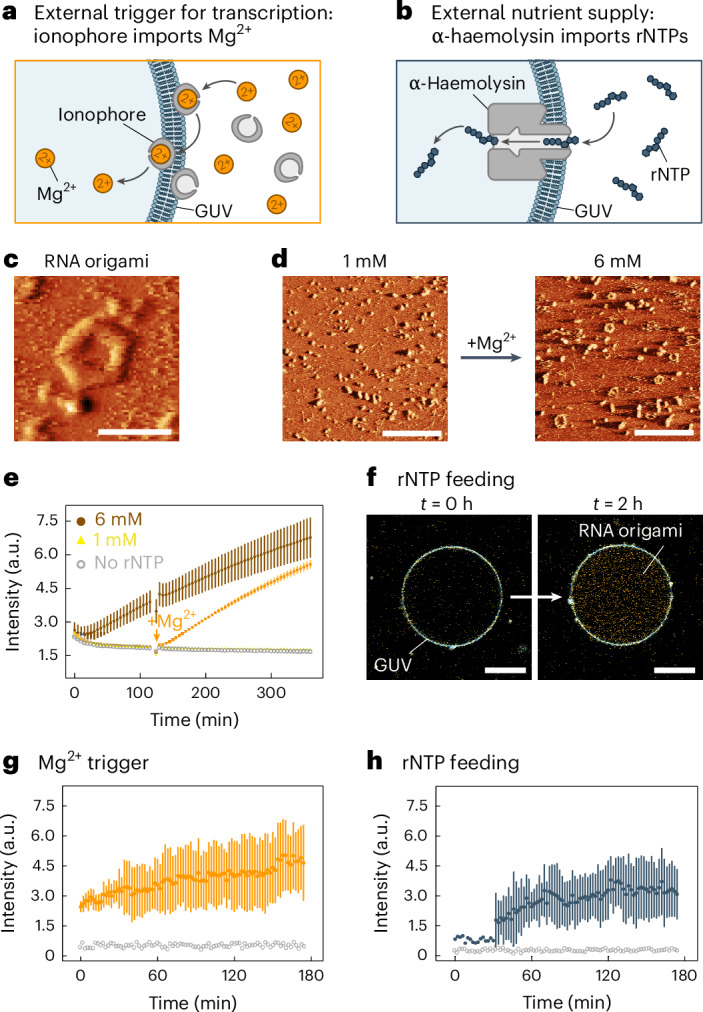
Expression of RNA origami inside GUVs. **a**,**b**, Schematic illustration showing the external triggers for RNA origami expression by Mg^2+^ import using ionophores (**a**) or rNTP feeding via α-haemolysin pores (**b**). **c**, AFM micrograph of the pentagon-shaped RNA origami (3H-4DT-iSpi), composed of five subunits with three helices each. Scale bar, 50 nm. **d**, AFM micrographs of Mg^2+^-triggered 3H-4DT-iSpi RNA origami. RNA was incubated for 2 h at 1 mM Mg(OAc)_2_ (left) before transcription was triggered by increasing Mg(OAc)_2_ to 6 mM and incubated for another 2 h (right). Scale bars, 200 nm. Panels **c** and **d** are images from lock-in phase channel. The corresponding height images are shown in Supplementary Fig. 1. **e**, Fluorescence plate reader experiments tracking 3H-4DT-iSpi RNA origami production by measuring DFHBI-1T emission over time (*λ*_ex_ = 488 nm, mean ± s.d., *n* = 3 wells). The orange arrow indicates the time point where the Mg(OAc)_2_ concentration was increased from 1 mM to 6 mM. **f**, Confocal overlay images of 3H-4DT-iSpi RNA origami (orange, iSpinach binds DFHBI-1T, *λ*_ex_ = 488 nm) transcribed inside of a GUV (blue, membrane labelled with DiD, *λ*_ex_ = 640 nm). Quantification in Supplementary Fig. 5. Scale bars, 20 μm. **g**,**h**, RNA origami transcription inside of GUVs triggered by addition of Mg^2+^ (**g**) or rNTPs (**h**) over time (mean ± s.d., *n* = 6 GUVs). The data were extracted from confocal fluorescence time-lapse recordings (Supplementary Videos 1 and 2). Empty grey circles denote the background fluorescent signal outside of the GUVs.

We chose a Mg^2+^ ionophore^26^ capable of shuttling Mg^2+^ across the membrane with high specificity (Fig. 2a). The ionophore gives us a membrane-protein-free closed system^27^; however, waste from the transcription process cannot escape the vesicle.

For combined Mg^2+^ import, nucleotide feeding and waste removal, we incorporated the commonly used bacterial transmembrane pore α-haemolysin. With a constriction of 1.4 nm, this pore allows us to continuously fuel transcription from a bulk feeding solution (containing rNTPs and Mg^2+^) while preventing the escape of the larger DNA template, RNA polymerase and RNA origami structures (Fig. 2b).

We initially chose a previously realized two-dimensional RNA origami that has a well-defined structure and attached a fluorescent iSpinach aptamer^28^ to the 3′ end (3H-4DT-iSpi, originally designed in ref. ^23^; Supplementary Fig. 1). The RNA origami folds into a 3-helix tile, which further assembles into a 5-tile pentagon with 20 nm edges (Fig. 2c). Experiments showed that 1 mM Mg^2+^ is insufficient for transcription (Fig. 2d, left), while we obtained the correctly folded RNA origami at 6 mM (Fig. 2d, right, Fig. 2e and Supplementary Fig. 1b). We verified these findings with another RNA origami design^23^ (Supplementary Fig. 2).

Next, we moved the transcription of the RNA origami into GUVs. The ionophore and Mg^2+^ were added to the external buffer after GUV formation. We tracked RNA origami production by observing iSpinach fluorescence inside individual GUVs with confocal microscopy (Fig. 2g, Supplementary Video 2 and Supplementary Fig. 3). The mean fluorescence intensity inside the GUVs increased consistently over time compared with the bulk solution, confirming successful RNA origami production.

To implement nucleotide feeding, we reconstituted α-haemolysin in the GUVs^29^. As all salts required for transcription could be supplied after formation, this strategy improved the GUV formation efficiency, which is sensitive to ions^30^. RNA origami production was successfully initiated by adding rNTPs and salts to the external buffer (Fig. 2f,h and Supplementary Video 1). A control experiment showed similar transcription patterns triggered with rNTPs in bulk solution (Supplementary Figs. 4 and 6–8 and Supplementary Video 3).

### Co-transcriptional folding of RNA origami nanotubes

Having successfully expressed RNA origami in GUVs, we set out to provide a proof of principle for the genetic encoding of RNA origami structures with cell-like functions. Inspired by the success of DNA-based mimics of cytoskeletons^16,31–35^, we wanted to realize genetically encodable RNA origami nanotubes. For our purpose, an RNA cytoskeleton has to fold co-transcriptionally and form stiff, cell-sized structures from a low number of genes (strands). Previous studies have demonstrated micrometre-scale one-dimensional^36^ and two-dimensional^37,38^ RNA filaments; however, they required thermal annealing and were not produced co-transcriptionally^39^. Moreover, microtubules, nature’s stiffest filaments, have a hollow three-dimensional (3D) architecture, which maximizes the trade-off between material and persistence length. In line with these criteria, we engineered single-stranded RNA tiles that were able to co-transcriptionally fold and assemble into micrometre-length nanotubes.

**Fig. 3:**
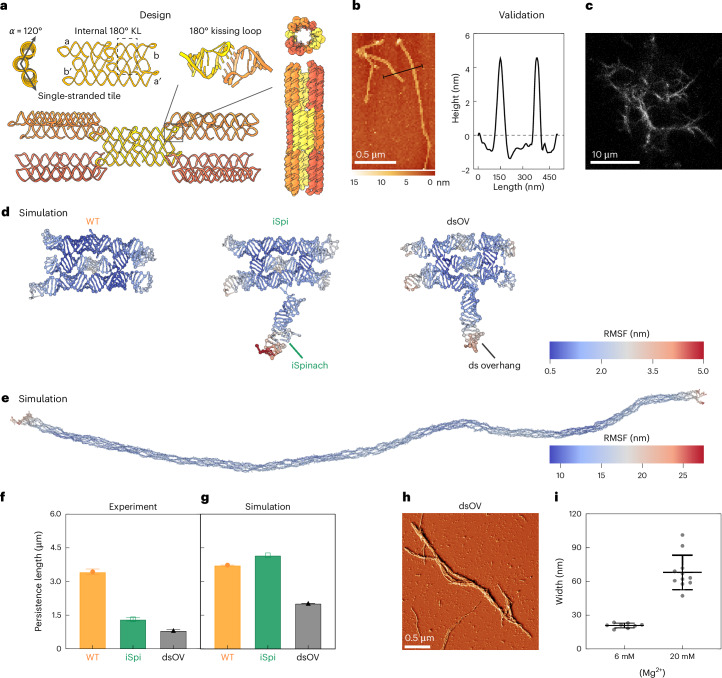
Design and characterization of cytoskeleton-like RNA origami nanotubes. **a**, In silico design of RNA origami nanotubes. The nanotube-forming RNA origami tiles assemble co-transcriptionally from a single RNA strand, forming 3 duplexes with *α* = 120° intrinsic curvature. The 180° external kissing loops (KLs) for nanotube assembly are paired corner-to-corner (a–a′ and b–b′). **b**, AFM micrograph of WT RNA origami nanotubes. The height profile is plotted along the black line. **c**, Confocal image of iSpi RNA origami nanotubes after 12 h of in vitro transcription. Fluorescence visualization was achieved by including an iSpinach aptamer and DFHBI-1T dye (*λ*_ex_ = 488 nm). **d**, oxRNA MD simulations of WT, iSpi and dsOV RNA origami tiles. **e**, oxRNA MD simulation of a 300-tile assembly (here WT). Panels **d** and **e** show the centroid structures from simulation. Each nucleotide is coloured by its RMSF. **f**,**g**, Persistence length of the RNA origami nanotubes calculated from AFM images (mean ± s.e. of fit; Methods; **f**) and simulations (300-tile nanotubes during the final 10% of the time steps, mean ± propagated s.e. of fit, *n* = 603 simulation frames; Supplementary Note 1; **g**). **h**, AFM micrograph (error signal mode) of bundled dsOV RNA origami nanotubes upon addition of Mg^2+^ as a crosslinker. The corresponding height image is shown in Supplementary Fig. 18. **i**, Width of non-bundled and bundled dsOV RNA origami nanotubes (mean ± s.d., *n* = 8 and 11 nanotubes, respectively).

The design process (Supplementary Fig. 9a) yielded a ‘wild-type’ (WT) tile with approximate dimensions of 11 nm × 5 nm × 2.5 nm forming a nanotube with an outer diameter of 11 nm (Fig. 3a and Supplementary Fig. 9b). The in silico design was then validated experimentally with atomic force microscopy (AFM). The co-transcriptionally folded tiles assembled into nanotubes up to several micrometres in length. AFM yielded an apparent nanotube height of 5–6 nm, which corresponds to at least two RNA duplexes lying on top of one another (an unravelled nanotube would have a height below 2 nm corresponding to a single RNA duplex) (Fig. 3b). At the same time, the measured width is ~22 nm (Supplementary Fig. 10). The deviations from the expected dimensions are consistent with the expected tip compression and surface collapse for a hollow nanotube design. Owing to strong kissing loop interactions, RNA nanotubes withstand temperatures up to 50 °C (Supplementary Fig. 11).

Next, we designed a tile with an iSpinach aptamer extending from the 3′ end (hereafter ‘iSpi’) (Supplementary Fig. 29) to visualize the RNA origami nanotubes with confocal microscopy. We obtained cytoskeleton-like networks tens of micrometres across (Fig. 3c). Compared with AFM, confocal microscopy was performed without removing the DNA template and the polymerase in a purification step. The addition of the relatively bulky iSpi aptamer does not influence the mean length of the RNA origami nanotubes (Supplementary Fig. 12).

Since the modification of individual tiles is key for the creation of functional cytoskeletons, we generated a third design modified with a double-stranded loop-out (hereafter ‘dsOV’). The loop-out sequence is connected to the tile via a three-way junction near, but not at, the 3′ end (Fig. 3d and Supplementary Fig. 29). The dsOV nanotubes feature slightly lower mean length (706.1 ± 563.8 nm versus 970.9 ± 735.1 nm for WT).

With coarse-grained molecular dynamics (MD) simulations, we simulated single tiles (Fig. 3d) as well as 1 μm (300-tile) nanotubes using the oxRNA model^40,41^. The 300-tile assemblies were built from relaxed monomers, resulting in simulations with over 90,000 nucleotides (Fig. 3e and Supplementary Fig. 13). To our knowledge, these are the largest structures simulated in oxRNA so far. We compared the persistence length of the nanotubes extracted from AFM images with that of simulated nanotubes (Fig. 3f,g). Experiments yielded a persistence length of 3.4 μm for WT nanotubes, while iSpi and dsOV exhibited lower persistence lengths (1.3 μm and 0.8 μm, respectively). The addition of dye did not impact the persistence length (2.6 μm for WT and 1 μm for iSpi; Supplementary Fig. 31).

Despite overestimating the persistence lengths of iSpi and dsOV nanotubes (4.2 μm and 2.0 μm, respectively), the MD simulations estimated the persistence length of the WT nanotubes almost correctly at 3.7 μm. The simulation also reproduced the fact that the dsOV tiles yield the least stiff nanotubes. Since oxRNA lacks the ability to model non-canonical base pairing and ions are represented implicitly, we do not expect to perfectly capture the effects of highly structured aptamers such as iSpinach. However, the results with the WT and dsOV structures give us confidence in using the coarse-grained simulations to explore the observed differences in stiffness with nucleotide-level resolution.

In the simulations of the dsOV nanotubes, we observe higher rates of bond-breaking, which are not seen in the WT design (Supplementary Figs. 14 and 15). This indicates that the lower persistence length of dsOV may result from assembly defects and fragmentation of assembled RNA origami nanotubes.

In experiments, the ratio of calculated persistence length to mean contour length, which indicates the flexibility of the nanotubes, was approximately 3.5 for WT, 1.6 for iSpi and 1.1 for dsOV. These results suggest that while the addition of a helix does not affect the length of the resulting nanotubes (Supplementary Fig. 12), it does impact their flexibility (Fig. 3f). We hypothesize that there are two major factors impacting nanotube stiffness, namely, steric hindrance during assembly and intrinsic tile flexibility in the assembled nanotubes (as discussed in Supplementary Note 1 and Supplementary Figs. 14–17).

It is known that flexibility impacts polymer aggregation. Flexible polymers experience negligible loss of entropy during dimerization, making bundling more favourable compared with semiflexible polymers^42^. Since bundling is key in cytoskeletal organization, we examined our nanotubes for their bundling capacity. We induced bundling in the highly flexible dsOV by increasing the Mg^2+^ concentration to 20 mM, comparable to cellular concentrations, which has previously been used to bundle DNA nanotubes^43^. We observed the formation of long nanotube bundles with a width of 67.91 ± 15.47 nm (Fig. 3h,i and Supplementary Fig. 18). By comparison, bundling was not seen for the semiflexible WT and iSpi designs (Supplementary Fig. 19).

### Formation of RNA origami rings

**Fig. 4:**
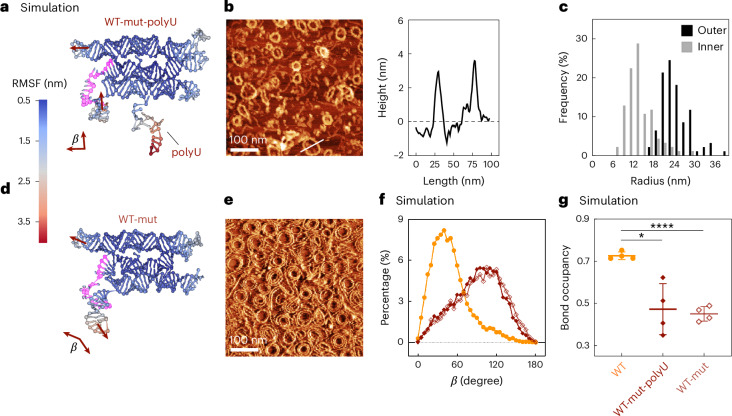
Formation of RNA origami rings. **a**,**d**, Coarse-grained MD simulation of a sequence-mutated WT tile (WT-mut) with (**a**) and without (**d**) single-stranded overhang polyU. All designs have GGAA starting sequence. The 12 U nucleotides 5′ overhang of WT-mut-polyU was intended for binding to a fluorescent probe. *β* denotes the angle between the upper left and lower left duplexes as shown. The broken stem-loop in the middle left helix is highlighted in pink. The centroid structure from simulation is shown. Each nucleotide is colour-coded according to its RMSF. **b**, AFM micrograph and corresponding height profile of in vitro-transcribed WT-mut-polyU RNA origami rings. The height profile is plotted along the white line. **c**, Histogram of the inner and outer radius of RNA origami rings (*n* = 94, calculated as half the Feret diameter). **e**, AFM micrograph (lock-in phase channel) of RNA origami rings from the WT-mut tile. The corresponding height image is shown in Supplementary Fig. 21b. **f**, Distribution of the angle *β* of simulated single tiles using oxRNA coarse-grained simulation (WT, orange circles; WT-mut-polyU, maroon-filled diamonds; WT-mut, light-maroon empty diamonds). **g**, Bond occupancy of the stem-loop highlighted in **a** and **d** (mean ± s.d., *n* = 4 simulation runs). A parametric, unpaired *t*-test with Welch’s correction was performed. Two-tailed *P* values of the comparison between WT and the mutated designs are marked with **** (*P* = 5.3 × 10^−5^) and * (*P* = 0.0234). The difference is not significant among the mutated designs (*P* = 0.7295).

During our exploration of RNA origami nanotube designs, we identified mutations on the DNA template that consistently formed RNA origami rings rather than nanotubes. The ring-forming design used the same strand routing as WT, however with a different sequence. It also contained a single-stranded, U-rich overhang at the 5′ end (hereafter WT-mut-polyU) (Fig. 4a). In oxRNA simulations, WT-mut-polyU consistently showed misfolding in the stem-loop on the ‘left’ side of the middle helix, which was not observed in WT, iSpi or dsOV (Fig. 4a, highlighted sequence, and Supplementary Fig. 20). This region in the new sequence has a GC (guanine cytosine) content of 50% GC compared with 75% GC in the other designs. The nanorings formed both co-transcriptionally (Fig. 4b) and via thermal annealing (Supplementary Fig. 21a), indicating that the misfolding is inherent to the sequence and not a kinetic trap. Notably, the height of the ring was lower than that of the nanotubes (4 nm versus 6 nm; Fig. 4b) and the rings showed a relatively homogeneous radius (23.44 ± 4.18 nm; Fig. 4c).

To confirm that the rings resulted from misfolding in the indicated region and not the overhang, we removed the overhang (hereafter WT-mut). We continued to observe misfolding in oxRNA simulations (Fig. 4d), while in experiments, WT-mut also formed nanorings after both thermal annealing (Fig. 4e) and co-transcriptional folding (Supplementary Fig. 21b). We characterized the angle, *β*, between the upper and lower left kissing loop (KL)-containing duplexes of WT-mut in single-tile simulations to assess the effect of misfolding on intertile connections (Fig. 4a,d). The WT, WT-mut-polyU and WT-mut designs exhibited an angle *β* of 49.50 ± 28.82°, 90.61 ± 35.62° and 94.29 ± 37.23°, respectively (Fig. 4f). These duplexes are intended to be parallel to facilitate polymerization into a straight nanotube; however, in the mutated designs, the misfolded stem-loop shifts the distribution to a perpendicular angle between the helices, which favours assembly into rings. Bond occupancy in this region in the single-tile simulations dropped from 0.73 ± 0.02 (WT design) to 0.47 ± 0.12 and 0.45 ± 0.03 (WT-mut-polyU and WT-mut, respectively) (Fig. 4g).

We validated the hypothesis that ring formation is caused by opening of the stem-loop experimentally by expressing and co-transcriptionally folding WT-mut tiles at 50 mM Mg^2+^, as divalent ion concentration enhances stiffness and hybridization in nucleic acids. As expected, the phenotype shifted towards short filaments of 3 nm in height and 81.14 ± 45.36 nm in length (Supplementary Fig. 22). This indicates that the ring formation was indeed dependent on the opening of the stem-loop. However, the length distribution peaked at 45 nm, less than half the length of an opened ring. This suggests additional intra-tile twist that hinders the growth of the filament.

While RNA nanorings as such have been demonstrated before^23^, the rings here arise from mutations in the tile design, demonstrating phenotypic plasticity of the system. With the same RNA tile blueprint, we have two distinctly different phenotypes.

### Expression of RNA origami cytoskeletons in synthetic cells

**Fig. 5:**
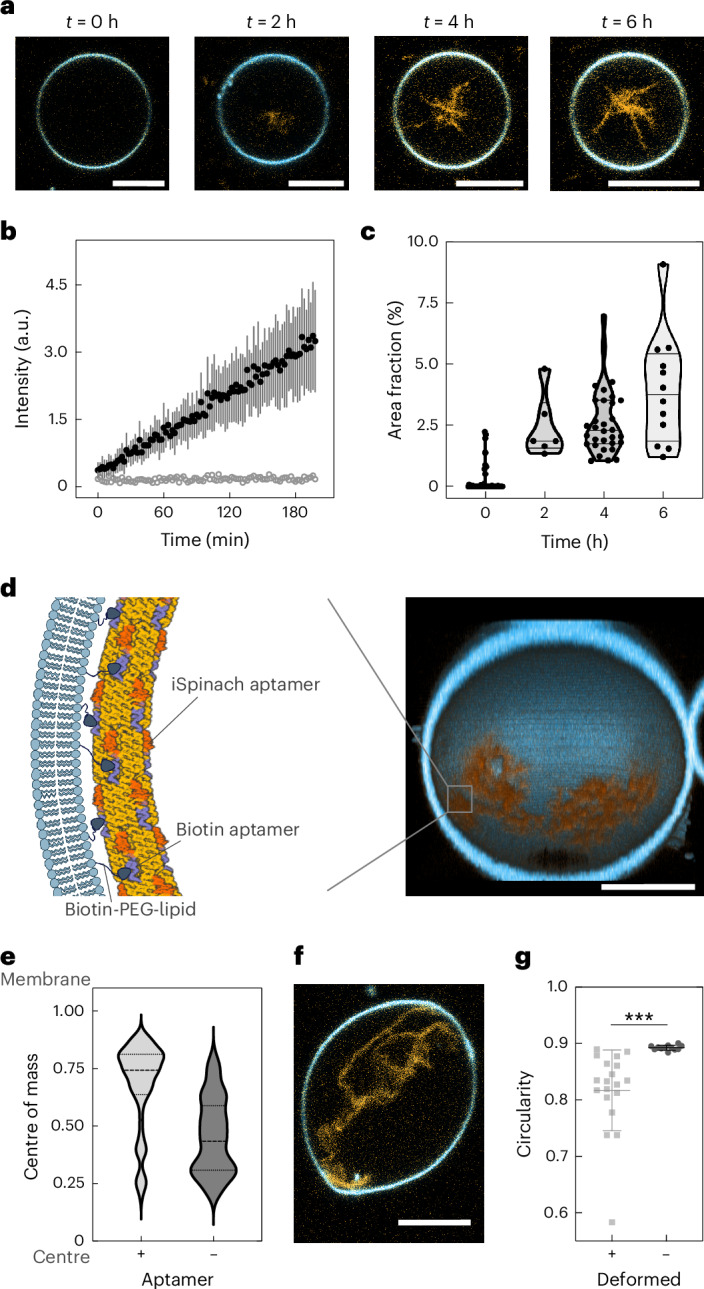
Expression of RNA origami cytoskeletons in synthetic cells. **a**, Confocal time series of cytoskeleton-like iSpi RNA origami nanotubes (orange, *λ*_ex_ = 488 nm) expressed inside a GUV (blue, membrane labelled with DiD, *λ*_ex_ = 640 nm). Scale bars, 10 μm. **b**, RNA nanotube transcription inside of GUVs triggered by addition of rNTPs plotted over the first 3 h of expression (mean ± s.d., *n* = 6 GUVs). The data were extracted from confocal fluorescence time-lapse recordings (Supplementary Video 4). Background fluorescence outside of the GUVs is plotted as grey circles. **c**, Area fraction occupied by the RNA origami nanotubes plotted over time (*n* = 50, 6, 32 and 12 GUVs, left to right). **d**, Cortex formation with RNA origami nanotubes on the inner GUV membrane. Left: schematic representation of an RNA origami nanotube adhering to the biotinylated GUV membrane via a biotin aptamer attached to the iSpi tile^38^. Right: confocal 3D reconstruction of an RNA origami cortex on the inner GUV membrane after its expression. Scale bar, 10 μm. **e**, Distribution of RNA nanotubes with or without the biotin aptamer inside of the GUV. The distribution of the centre of mass of the iSpi fluorescence relative to the GUV centre is plotted (*n* = 78 (+) and 87 (−) GUVs; median, dashed lines; first and third quartile, dotted lines). **f**, GUV deformation caused by biotin aptamer-functionalized RNA origami nanotubes. Scale bar, 10 μm. **g**, Quantification of GUV deformation. The circularity of deformed GUVs (*n* = 19) and GUVs not expressing nanotubes (*n* = 12) are plotted (mean ± s.d.). A parametric, unpaired *t*-test with Welch’s correction was performed. Two-tailed *P* value is marked with *** (*P* = 0.0002).

The cytoskeleton plays a crucial role in regulating cell shape and mechanics. Having built cytoskeleton-like RNA nanotubes that can fold co-transcriptionally, our next goal was to express these structures inside GUVs. Transcription of the iSpi tile inside GUVs was triggered by supplying 4 mM rNTPs externally via α-haemolysin pores and monitored with confocal microscopy, showing the appearance of a network of RNA origami nanotubes over the course of 6 h (Fig. 5a and Supplementary Video 4). Both mean fluorescence intensity (Fig. 5b) and area fraction of the cytoskeleton network within the GUVs increased over time (Fig. 5c, Supplementary Videos 5, 6 and 7, and Supplementary Fig. 23).

Inspired by septin, a natural membrane-binding cytoskeleton component^44^, we aimed for cortex formation as the first function of our RNA cytoskeleton. To establish binding between RNA nanotubes and the vesicle membrane, we added a biotin aptamer to the iSpi tile (Fig. 5d, left, and Supplementary Fig. 24a). We expressed these tiles in GUVs with 5% biotinylated lipids. When transcription was triggered with rNTPs, the RNA nanotubes containing iSpinach and biotin aptamers assembled into a cortex on the inner GUV membrane (Fig. 5d, right, and Supplementary Video 8). RNA origami nanotubes were exclusively observed bound to the biotinylated membrane in the presence of the biotin aptamer (Supplementary Fig. 24b). Without the biotin aptamer, RNA origami nanotubes were observed in the GUV lumen as in previous experiments (Supplementary Fig. 24c). We quantified membrane binding by calculating the centre of mass of the fluorescence radial distribution in the expressing GUVs. Membrane binding results in a shift in the centre of mass of the RNA fluorescence signal towards the GUV membrane (Fig. 5e and Supplementary Fig. 24d,e). Interestingly, continuous supply of nucleotides resulted in high expression levels in some GUVs, which, in turn, caused GUV deformation both with and without biotin aptamers (Fig. 5f and Supplementary Figs. 25–27). Note that owing to the stochasticity of the GUV encapsulation process of polymerase, DNA template or nanopores, deformation is observed in only about 5% of the RNA nanotube-producing GUVs, but never in non-expressing GUVs (Supplementary Fig. 28). We quantified this deformation by measuring the circularity of the deformed GUVs compared with non-expressing control GUVs (Fig. 5g). The deformation is statistically significant, reducing the circularity to a mean value of 0.8. Deformation happens primarily along the axis of the RNA origami cytoskeleton inside the GUV (examples of deformed GUVs shown in Supplementary Figs. 25–27 and Supplementary Videos 9 and 10), suggesting an active deformation by polymerization rather than osmotic deflation. Osmolarity changes during the experiment are negligible (within the μM range; Supplementary Note 2).

## Conclusion

Here we provide a conceptual framework for the construction of synthetic cells based on the expression of RNA origami inside of lipid vesicles. Compared with DNA origami, RNA is genetically encodable, which means that the synthetic cells can produce their own building blocks in an active, out-of-equilibrium process. This makes the approach different from the mere encapsulation of DNA origami structures. In addition, we have shown that we can incorporate functionality, such as membrane binding, using RNA aptamers instead of covalent modification with, for example, cholesterol needed on DNA structures. Finally, subtle changes to the design, such as simply swapping out overhanging sequences or weakening specific helices, can result in substantial morphological changes, hinting towards exciting prospects for directed evolution approaches. Compared with proteins, RNA origami expression in GUVs required far fewer biologically derived components—only T7 polymerase compared with the entire transcription–translation machinery with over 150 genes. This could mean that the realization of a self-sustained chemical system capable of evolution becomes more accessible^45,46^—although this remains speculative for now and diverse scientific communities may, one day, realize diverse variants of synthetic cells.

We expect future RNA origami-based hardware to incorporate ribozymes for the realization of molecular machines, which execute complex cellular tasks. From a more fundamental point of view, the study of ribozyme activity in confinement has been shown to deviate from bulk activity^47,48^, which hints towards exciting prospects for active RNA origami in vesicles. In addition, it will be interesting to develop methods to copy the DNA template, for example, by templated ligation^49^, and the use of polymerase ribozymes^50^ instead of T7 for RNA production or direct self-replication of RNA origami. Notably, the construction of synthetic cells based on co-transcriptional RNA origami is inherently compatible with evolution and AI-guided design, which allows orders of magnitude higher throughput and design testing compared with traditional rational engineering approaches. This will increase the functional complexity of synthetic cells while at the same time incorporating evolution, as a fundamental characteristic of life, into the design process itself. From a practical point of view and beyond synthetic life, genetically encodable hardware made from RNA origami may lead to new exciting biophysical tools to manipulate cells and to address questions in cell biology.

## Methods

### Design of RNA origami

The design of the RNA tiles was guided by the principles established by Geary et al. for ssRNA origami^24^.

The 3H-4DT-iSpinach blueprint was generated by adding the iSpinach module to the 3′ end of the 3H-4DT design from Geary et al.^23^. The iSpinach aptamer was connected to the core design by a 2 uracil linker.

The blueprints for the nanotube-forming tiles were designed based on the 3H-3DT tile from Geary et al.^23^. The first and third helices were extended so that the helical turn of the external 180° kissing loops would align upon assembly. This extension process was done manually via cycles of extension and visualization using the RNAbuild script from ROAD^23^ and ChimeraX^51^ to minimize the distance between kissing loops upon closure of the tube. During this process, the length of the helices were also adjusted so that the internal kissing loops would correspond to 8 base pair duplexes instead of the original 9 in the 3H-3DT design, reflecting the updated understanding of internal kissing loop geometry based on cryoEM structures^52^.

The blueprint of the iSpi design was then put into Revolvr^23^. The final sequence was chosen using two criteria: both external kissing loop pairs should have binding energy below −8 kcal mol^−1^, and the difference in binding energy between the two pairs should be minimal. The WT design was created by removing the iSpi overhang. The dsOV was introduced into the tile blueprint without further sequence optimization. All blueprints were used as input for the trace_analysis script from ROAD^23^ to check for folding irregularities and then used for oxRNA simulations.

The blueprints of the mutated ring-forming tiles were generated as described, and the sequence optimization was performed in the same manner described for the other tiles.

The RNA origami containing biotin aptamer was designed by concatenating the WT, biotin aptamer and iSpinach aptamer in the following sequence: WT tile-AAA-biotin aptamer-AAAA-iSpinach aptamer (Supplementary Fig. 29). The sequence was then checked using the RNAfold web server^53^ to ensure correct folding of each aptamer in the concatenated sequence.

### Synthesis of RNA origami in bulk

DNA templates were synthesized as double-stranded gBlocks from Integrated DNA Technologies (IDT). DNA gBlocks (0.5 ng μl^−1^) were PCR-amplified using 14–25-nt primers (Supplementary Data 1), using a Phusion High-Fidelity PCR Kit (NEB) with annealing temperature at 62 °C. The PCR product was then purified using a Qiagen PCR purification kit. RNA was transcribed and co-transcriptionally folded in a one-pot reaction containing PCR purified DNA template (4 ng μl^−1^), Mg(OAc)_2_ (6 mM), NaOAc (40 mM), KCl (40 mM), Tris-OAc (50 mM, pH 7.8), rNTPs (1 mM each), dithiothreitol (DTT) (1 mM) and DFHBI-1T dye (62.5 μM) and RNase inhibitor (1 U μl^−1^), if not described otherwise. Reactions were initiated by adding T7 RNA polymerase (0.2 U μl^−1^). Transcription reactions were carried out in 100 μl volumes at 37 °C for 2–12 h depending on the experimental setup. All the experiments were performed using nuclease-free water.

### Fluorescence assay in bulk

3H-4DT RNA origami with an iSpinach fluorophore was prepared with PCR purified DNA template (4 ng μl^−1^), NaOAc (40 mM), KCl (40 mM), Tris-OAc (50 mM, pH 7.8), rNTPs (1 mM each), DTT (1 mM) and DFHBI-1T dye (62.5 μM) in four different conditions of Mg(OAc)_2_ (that is, 0 mM, 1 mM, 1–6 mM and 6 mM). In the 1–6 mM sample, first, 1 mM Mg(OAc)_2_ was added for 2 h and then up to 5 mM of Mg(OAc)_2_ added for the next 2 h. To measure the fluorescence intensity, 100 μl final volume was pipetted into a Greiner 96-well black bottom plate (Sigma-Aldrich), which was finally placed inside the plate reader (Spark multimode plate reader from Tecan Life Sciences) for 4 h at 37 °C. RNA production was quantified by measuring the emission of DFHBI-1T upon excitation with 488 nm (DFHBI-1T dye, *λ*_ex_ = 482 nm, *λ*_em_ = 505 nm) with a gain of 100 (manually set).

### GUV preparation

GUVs were prepared by the polyvinyl alcohol (PVA) gel-assisted swelling method^27,29^. In detail, a PVA solution was prepared by mixing 5% (w/v) PVA (MW 145,000 Da) in nuclease-free water with sucrose (100 mM) for 24 h, at 400 rpm, at 90 °C. The PVA solution (50 μl) was dried as a thin film on a glass slide (60 mm × 24 mm) at 50 °C for 30 min. Then, 5 μl of a lipid mixture in chloroform containing 10 mol% DOPG (10 μg μl^−1^) and 1 mol% DiD dye in 10 μg μl^−1^ DOPC was spread onto the PVA layer and dried for 1 h at 37 °C. The DiD dye stock solution (10 μg μl^−1^) was prepared in chloroform. Using a Teflon chamber (approximately 40 mm × 24 mm) as a spacer and a second glass slide, a chamber was assembled on top of the slide with the lipid-coated PVA. Then, the lipids were hydrated with 1 ml of 100 mM sucrose containing all the transcription components to be loaded into the GUV depending on the experiment for 1 h at room temperature, allowing for GUV formation. After that, the chamber was inverted for 5 min and gently tapped twice using a pipette tip, and the GUVs were collected into a 1.5 ml Eppendorf tube and left to settle for 30 min. For the washing, 350 μl of GUVs was taken from the bottom and added to 1 ml of 150 mM glucose buffer. The GUVs were allowed to settle overnight at 4 °C. The next day morning, the top 1 ml of the buffer was removed without disturbing the bottom layer. The GUVs were washed second time with 0.5 ml of 150 mM glucose buffer, and the GUVs were only allowed to settle for 2–3 h at 4 °C. For subsequent experiments, the GUV solution was sourced from the bottom, where there was a higher likelihood of obtaining GUVs with effectively encapsulated DNA templates and RNA polymerase, attributable to their greater density. All experiments were performed using nuclease-free water. For imaging, an 18-well Ibidi glass bottom chamber was pre-incubated with 3% BSA solution and washed with nuclease-free water twice before GUV addition.

### Expression of RNA origami triggered by Mg^2+^ in GUVs

GUVs containing a mixture of the DNA template (4 ng μl^−1^), RNA polymerase (0.2 U μl^−1^), each rNTP (1 mM), Mg(OAc)_2_ (1 mM), NaOAc (40 mM), KCl (40 mM), Tris-OAc (50 mM, pH 7.8), DTT (1 mM), DFHBI-1T dye (62.5 μM) and sucrose (150 mM) were formed as described. Subsequently, a specific washing protocol was used, wherein 350 μl of GUVs was rinsed once with 1 ml of 570 mM glucose buffer, maintained at room temperature for 2–3 h. On the same day, 100 μl of the washed GUV solution was transferred to an 18-well imaging chamber and supplemented with 10 µM magnesium-ionophore I (Merck). Once the sample was placed on the confocal microscope with an incubation chamber held at 37 °C for 2 h. Following this incubation, an additional 5 mM of Mg(OAc)_2_ was introduced into the GUV solution. The GUVs were continuously monitored for up to 4 h.

### Expression of RNA origami triggered by rNTPs in GUVs

GUVs containing a mixture of the DNA template (4 ng μl^−1^), RNA polymerase (0.2 U μl^−1^) and α-haemolysin (15 ng μl^−1^) were formed as described. On the following day, 80 μl of purified GUVs was supplemented with a feeding solution containing sucrose (100 mM), Mg(OAc)_2_ (6 mM), NaOAc (40 mM), KCl (40 mM), Tris-OAc (50 mM, pH 7.8), DTT (1 mM) and DFHBI-1T dye (62.5 μM). These components could enter the GUV lumen via the α-haemolysin pores. The GUVs, now containing the necessary components for co-transcriptional folding except rNTPs, were incubated for 2 h at 37 °C. They were then transferred to an 18-well imaging chamber and allowed to settle to the bottom of the slide for 20 min before imaging commenced. Once the sample was placed on the confocal microscope with an incubation chamber held at 37 °C, rNTPs were added externally to achieve a final concentration of 1 mM for each nucleotide. The rNTPs also translocate into the GUVs via α-haemolysin. GUVs were monitored over a period of up to 4 h. Alternatively, to image the formation of the RNA origami cytoskeleton-like at discrete time points (0 h, 2 h, 4 h and 6 h), GUVs were incubated with all above-mentioned components including rNTPs in an Eppendorf tube within a thermal block set to 37 °C before imaging.

### oxRNA simulations

Coarse-grained modelling of individual RNA tiles and the nanotubes was performed using the oxRNA2 force field^40,54^ with the CUDA-accelerated oxDNA MD simulation engine (version 3.5.2)^41,55,56^. Structures were exported from ROAD schematics to PDB format using the RNAbuild script from ROAD^23^. The PDB structures were then converted to oxDNA simulation files using a local copy of TacoxDNA updated to work with Python 3.11 (ref. ^57^) (pipeline script in Code availability). Structures were relaxed using the protocol detailed in refs. ^58,59^. In brief, a short Monte Carlo simulation was performed to remove excluded volume clashes between nucleotides, followed by a longer (at least 5 × 10^7^ steps, *dt* = 0.003) MD relaxation performed using the ‘Langevin’ thermostat and a modified backbone FENE potential to avoid numerical instabilities owing to high forces. Harmonic traps (stiff = 3.1) were placed between all paired nucleotides to maintain the structure during relaxation. Successful relaxation was verified by observing per-nucleotide energy values below −1.4 and visualization of the intended structure with oxView^59,60^.

Multiple production equilibrium MD runs were performed on NVIDIA A100 GPUs using edge-based calculation parallelization^56^ for 1 × 10^9^ simulation steps at *dt* = 0.003. This roughly corresponds to a time step of 9.09 fs and a total simulation time of 9.09 μs by direct unit conversion. It should be noted, however, that calculating exact time correspondence in coarse-grained simulations is not straightforward. On the basis of a comparison between DNA binding rates in experiments and oxDNA simulations (see Supplementary Information of ref. ^61^), the time represented by these simulations may be closer to 30 ms. The temperature was maintained at 37 °C using an Andersen-like thermostat (thermostat = ‘brownian’ or ‘john’ in the oxDNA parameter file)^62^. Debye–Huckel electrostatics were applied to the system mimicking 0.5 M NaCl^54^. Configurations were saved every 5 × 10^5^ steps, resulting in trajectories with 2,000 frames each for analysis. Since the dynamics of correctly folded structures are of interest, we ran multiple simulations and selected the first four simulations where the internal kissing loops maintained a minimum of 11 out of designed 12 bonds on average throughout the simulation for further analysis.

The mean structures, root-mean-square fluctuations (RMSFs) and bond occupancies were calculated from simulation trajectories using oxDNA Analysis Tools (OAT, version 2.3.5)^60^. The structure closest to the mean structure was extracted from one replicate for each structure using the centroid function in OAT, and this structure was used to build the nanotube simulations using a custom-written Python script (Code availability); we built 100-layer (300 tiles, ~90,000 nucleotides) simulations from each single tile. The nanotubes were then relaxed with externally applied harmonic traps to ensure that the kissing loop cohesion was successfully formed. Once over 90% of target bonds were formed, the forces were dropped and triplicate production simulations with the same parameters as the single tiles were performed.

### Persistence length analysis

All AFM images were pre-processed with the open-access software Gwyddion^63^ using standardized Align Rows and Levelling tools and then exported into Tagged Image File Format (TIFF). The nanotubes were manually masked using Object Selection Tool and other Selection Tools in Adobe Photoshop 2024 version 25.12.0. The background was then removed and exported as a TIFF file. Owing to branching of the nanotubes, the background-removed images were then traced using a custom-written Python script (Code availability) that skeletonized the pre-selected nanotubes and traced along the longest path in case of branching (see analysis pipeline and Supplementary Fig. 30 in Supplementary Note 1).

The extracted coordinates were then used as input for a custom-written Python script (Code availability) to calculate the persistence length of the in vitro-transcribed nanotubes. To reduce detection error owing to low resolution in imaging, the data were filtered to exclude all detection under 200 nm. The squared end-to-end distance of each nanotube was plotted against its contour length. The data were then fit using the theoretical relation

1 $${\langle {R}^{2}\rangle }_{3D}=2sPL\left(1-\frac{sP}{L}(1-{e}^{-L/sP})\right)$$

where *R*^2^ is the squared end-to-end distance, *L* is the contour length, *P* is the persistence length and *s* is a surface parameter that is set to 1 (Supplementary Note 1). For each nanotube, the end-to-end distance and the total contour length of the nanotube were extracted for fitting. The data were masked by residuals ±1 s.d. For the simulated structures, owing to the narrow distribution of contour length (as the simulation is only one nanotube over time), one persistence length is calculated for each frame. The end-to-end distance of every layer pair and their contour lengths were fitted using the same equation and masking as described above. The data plotted in Fig. 3g are the last 10% of the simulation frames (*t* ≥ 9 × 10^8^).

### Analysis of RNA origami nanotube growth

Images of nanotube-producing GUVs at different time points (Fig. 5c) were analysed in ImageJ2 (ImageJ2 2.14.0/1.54f (ref. ^64^)) using custom-written ImageJ macro scripts (Code availability). In summary, for time point *t* = 0 h, the GUVs were automatically detected using the Hough Circle Transform plug-in. For further time points, the GUVs that produced RNA nanotubes were manually chosen. The regions of interest (ROIs) in the RNA fluorescence channel were then filtered and thresholded using the Gaussian Blur, Convert to Mask and Erode functions. The area fraction was then extracted for each GUV using the Measure function. For time point 0, false detections were manually removed. For other time points, only GUVs containing more than 1% area fraction were taken into account.

### Analysis of RNA cytoskeleton attachment to GUV membranes

Images of single GUVs that produced RNA cytoskeleton with and without biotin aptamer were analysed in ImageJ2 (ImageJ2 2.14.0/1.54f) using custom-written ImageJ macro scripts (Code availability). The GUVs were automatically detected using the Analyze Particles method. In short, the lipid fluorescence channel was processed using the Auto Threshold, Fill Holes, Erode, Gaussian Blur and then Convert to Mask functions before particle analysis. The detected ROIs were then manually filtered to remove false detection of non-GUV particles. The radial profile of each selected ROI was extracted using the Radial Profile function. The origin of the radial profile was the measured centroid of the ROI. The radius is the primary axis of a fitted ellipse to the ROI.

The radial profile was then analysed and plotted using a custom-written Python script (Code availability). The centre of mass of the RNA fluorescence relative to the GUV centre was calculated for each GUV using the formula

2 $${x}_{\mathrm{c}}=\frac{\mathop{\sum }\nolimits_{i = 1}^{N}{x}_{i}{y}_{i}}{\mathop{\sum }\nolimits_{i = 1}^{N}{y}_{i}}$$

where *x*_c_ is the centre of mass of the radial fluorescence, *x*_*i*_ are the normalized distance from the centre of the GUV, *y*_*i*_ is the normalized RNA radial fluorescence at distance *x*_*i*_ and *N* is the number of *x*, *y* pairs.

### GUV deformation

For all the deformation characterization, GUVs were encapsulated with DNA template (4–8 ng μl^−1^) with or without biotin aptamer, RNA polymerase (0.2–1 U μl^−1^), α-haemolysin (15 ng μl^−1^) and sucrose (200 mM). The GUVs were washed twice as mentioned above using a glucose solution (250 mM). On the next day, 80 μl of purified GUVs was supplemented with a feeding solution, either containing a homemade buffer consisting of Mg(OAc)_2_ (6 mM), NaOAc (40 mM), KCl (40 mM), Tris-OAc (50 mM, pH 7.8), DTT (1 mM) or a commercial 1× transcription buffer from Thermo Scientific (catalogue number EP0112) and DFHBI-1T dye (62.5 μM). To initiate the transcription process, GUVs were incubated for 6–12 h with 4 mM rNTPs in an Eppendorf tube within a thermal block set to 37 °C before imaging. For the control experiment, the same set-up was used without the addition of rNTPs.

### Analysis of deformation of GUVs owing to RNA nanotubes

Membrane detection and angular RNA origami nanotube intensity measurements were carried out with a custom-written ImageJ macro using ImageJ2 version 2.14.0/1.54f and afterwards analysed and plotted using a Jupyter Notebook (Python 3.10.13) (Code availability). For membrane detection, the membrane channel of the confocal microscopy images was processed using Gaussian Blur with *σ* = 3 and auto-thresholded using the Li method. The Convert to Mask, Fill Holes, Erode and Analyze Particle functions were then applied to obtain an ROI. The binary ROI image was saved in the output folder and the centroid coordinates of the ROI were calculated. The ROI images were then used to measure circularity using the ImageJ Measure function (set for Shape descriptors) where the circularity was defined as

3 $$R=\frac{4\pi A}{{P}^{2}}$$

where *A* is the area of the ROI and *P* is the perimeter of the ROI. For the GUVs where a z-stack was acquired, only the mean circularity is shown in Fig. 5g.

The distance for each coordinate in the ROI after membrane detection to the centroid is calculated and saved in a result file. To detect the RNA origami nanotubes, a second, rectangular ROI was drawn from the centroid towards the membrane with a length equal to 80% of the distance between the centroid and the membrane. The rectangular ROI was drawn for every 10th point on the membrane and the rectangle width was set to 3 μm. The mean intensity for each rectangle was measured and saved in another file. The Jupyter Notebook script was then used to load the data from the two files and extract and plot the radii and intensities.

### Statistics and reproducibility

Statistical analyses were conducted using a parametric, unpaired *t*-test with Welch’s correction. Data are presented as the mean ± s.d. from at least 6–78 GUVs observed with confocal microscopy, depending on the specific experimental conditions. For visualization, data in Fig. 2g,h were all plotted to 174 min. Persistence length values are presented as fitted value ± error of fit. Persistence length analysis was performed solely on co-transcribed nanotubes that were longer than 200 nm. Persistence length analysis was done on the middle 80% layer of each simulated nanotube with overhangs excluded from the analysis. Confocal and AFM experiments were each performed with more than six and two independent biological replicates, respectively. For plate reader assays, measurements were conducted in independent triplicates. Simulation data were generated from three independent runs. Only simulations of single tiles where the internal kissing loops contain more than 11 out of 12 base pairings were used for further in silico assembly and analyses. All analyses of nanotubes inside GUVs were conducted only on GUVs containing nanotubes. Empty GUVs were excluded from the analysis. No statistical method was used to predetermine sample size. The experiments were not randomized. The investigators were not blinded to allocation during experiments and outcome assessment.

### Data visualization and analysis

Plot graphing and statistical tests were performed using GraphPad Prism (version 5 and version 10.2.3) and Matplotlib 3.9.0.

### Reporting summary

Further information on research design is available in the Nature Portfolio Reporting Summary linked to this article.

## Online content

Any methods, additional references, Nature Portfolio reporting summaries, source data, extended data, supplementary information, acknowledgements, peer review information; details of author contributions and competing interests; and statements of data and code availability are available at 10.1038/s41565-025-01879-3.

## Supplementary information

Supplementary Information Supplementary Methods, Figs. 1–31, Notes 1 and 2, description of Supplementary Data 1 and Videos 1–10.

Reporting Summary

Supplementary Data 1 All the DNA templates and primers sequences are provided in an Excel sheet appended as Supplementary Data 1.

Supplementary Video 1 Transcription of 3H-4DT-iSpi RNA origami triggered by rNTPs. This confocal time-lapse captures the expression of 3H-4DT-iSpi RNA origami within GUVs upon addition of rNTPs. GUVs contained the DNA template, T7 RNAP and an α-haemolysin pore, which was used to transport the feeding solution (Methods) along with 6 mM Mg^2+^ except rNTPs for 2 h. The video begins after addition of 4 mM rNTPs and follows RNA origami production for 4 h at 37 °C.

Supplementary Video 2 Transcription of 3H-4DT-iSpi RNA origami triggered by Mg^2+^. This confocal time-lapse captures the expression of 3H-4DT-iSpi RNA origami within a GUV upon addition of Mg^2+^. GUVs contained the DNA template, T7 RNAP, the feeding solution (Methods) along with 4 mM rNTPs and 1 mM Mg^2+^. Ionophore (10 μM) was added externally and incubated for 2 h. The video begins after addition of 5 mM Mg^2+^ and follows RNA origami production for 4 h at 37 °C.

Supplementary Video 3 Transcription of S2T RNA origami triggered by rNTPs. This confocal time-lapse captures the expression of S2T RNA origami within GUVs upon addition of rNTPs (Supplementary Video 1). The video begins after addition of 4 mM rNTPs and follows RNA origami production for 4 h at 37 °C.

Supplementary Video 4 Transcription of iSpi RNA origami tiles triggered by rNTPs. This confocal time-lapse captures the expression of iSpi RNA origami tiles within GUVs upon addition of rNTPs (Supplementary Video 1). The video begins after addition of 4 mM rNTPs and follows RNA origami production for 4 h at 37 °C.

Supplementary Video 5 Transcription and assembly of RNA origami nanotubes triggered by rNTPs. This confocal time-lapse captures the formation of RNA origami nanotubes within a GUV upon addition of rNTPs (Supplementary Video 1). The video begins 2 h after addition of rNTPs at 37 °C.

Supplementary Video 6 Brownian motion of RNA origami nanotubes inside GUVs. These confocal time-lapses capture the fast movement of RNA origami cytoskeletons within GUVs. The videos were recorded after 6 h of transcription triggered by addition of rNTPs (Supplementary Video 1) for 21 s. The movement of the RNA cytoskeleton owing to Brownian motion is visible.

Supplementary Video 7 Brownian motion of RNA origami nanotubes inside GUVs. These confocal time-lapses capture the fast movement of RNA origami cytoskeletons within GUVs. The videos were recorded after 6 h of transcription triggered by addition of rNTPs (Supplementary Video 1) for 13 s. The movement of the RNA cytoskeleton owing to Brownian motion is visible.

Supplementary Video 8 RNA origami cortex formation on the inner membrane leaflet of GUVs. This confocal z-stack captures the biotin aptamer containing RNA origami nanotubes adhering to the biotinylated GUV membrane. RNA origami was expressed inside the GUV for 4 h at 37 °C before the z-stack was taken. The z-projection and 3D construction of this z-stack is shown in Supplementary Fig. 24b and Fig. 5d.

Supplementary Video 9 GUV with or without deformation owing to RNA nanotube-mediated cortex formation. This confocal time-lapse captures a deformed, biotinylated GUV containing RNA nanotubes with biotin aptamer (bottom) and another round, non-deformed biotinylated GUV (top) without RNA nanotube expression in the same microscopic field of view. RNA origami was expressed inside the GUVs for 6 h at 37 °C before the video was taken. The analysis of the deformed GUV is shown in Supplementary Fig. 25 (second GUV from the top) and Supplementary Fig. 26 (second GUV from the top left).

Supplementary Video 10 Z-stack of multiple GUVs deformed owing to RNA nanotube-mediated cortex formation. These confocal z-stacks capture RNA origami nanotubes with biotin aptamer adhering to the inner surface of the biotinylated GUV membrane. RNA origami was expressed inside the GUVs for 6 h at 37 °C before the z-stack was taken. The analysis of these GUVs is shown in Supplementary Fig. 25.

## Data Availability

Supporting data are available in the Supplementary Information. All raw data and analysis files used in the study are deposited on HeiData (10.11588/data/LJLAMX).

## Code availability

All custom-written codes were deposited on GitHub at https://github.com/Biophysical-Engineering-Group/RNAcytoskeleton.
