# Supplementary material for: Genetic encoding and expression of RNA origami cytoskeletons in synthetic cells
Source: Nat Nanotechnol. 2025 Mar 17;20(5):664–71. doi: 10.1038/s41565-025-01879-3 (PMC12095062; doi:10.1038/s41565-025-01879-3)
Supplement: Supplementary file 1 — Supplementary Methods, Figs. 1–31, Notes 1 and 2, description of Supplementary Data 1 and Videos 1–10. [file 41565_2025_1879_MOESM1_ESM.pdf]

nature nanotechnology

Supplementary information

https://doi.org/10.1038/s41565-025-01879-3

Genetic encoding and expression of RNA origami cytoskeletons in synthetic cells

In the format provided by the authors and unedited

Contents

1 Supplementary Methods **2**

1.1 Materials . . . 2

1.2 Atomic force microscopy . . . 2

1.3 Confocal Microscopy . . . 2

1.4 Analysis of RNA origami expression in GUV . . . 3

1.5 RNA origami bundling . . . 3

2 Supplementary Figures **3**

2.1 Expression of RNA origami inside GUVs (Figs. S1-S8) . . . 3

2.2 Construction of RNA origami nanotubes (Figs. S9-S19) . . . 10

2.3 Formation of RNA origami rings (Figs. S20-S22) . . . 17

2.4 Expression of an RNA origami cytoskeleton in synthetic cells (Figs. S23-S28) . . . 18

2.5 RNA origami blueprints . . . 24

3 Supplementary Data 1: Sequences **25**

4 Supplementary Notes **25**

4.1 Persistence length analysis . . . 25

4.2 Solution osmolarity . . . 27

5 Supplementary Videos (Videos 1-10) **27**

1 Supplementary Methods

1.1 Materials

DOPC(1,2-dioleoyl-*sn*-glycero-3-phosphocholine, cat #850375), DOPG(1,2-dioleoyl-*sn*-glycero-3-phospho-(1'-*rac*-glycerol) (sodium salt), (cat #840475) in chloroform were purchased from Avanti Polar Lipids. The membrane dye DiD (1,1'-Dioctadecyl-3,3,3',3'-Tetramethylindodicarbocyanine, 4-Chlorobenzenesulfonate salt, cat #D7757) and T7 RNA polymerase (cat #EP0112) were purchased from Thermo-Fisher Scientific. Sodium acetate (cat #S2889), Potassium chloride (cat #P3911), Sodium hydroxide (cat #S5881), Polyvinyl alcohol (cat #S8062894), DL-Dithiothreitol (cat #DO632), Sucrose (cat #S0389), Tris-acetate (cat #T1258), Glucose (cat #G6152), Magnesium-Ionophor I (N,N'-Diheptyl-N,N'-dimethyl-1,4-butandiamid)(cat #63082) and α-hemolysin from Staphylococcus aureus (cat #H9395) were purchased from Sigma-Aldrich. Magnesium acetate tetrahydrate (cat #A322119) was purchased from Merck. Phusion® High-Fidelity PCR Kit (cat #E0553S) and RNase Inhibitor, Murine (cat #M0314) were purchased from New England Biolabs. Nuclease free water was purchased from Integrated DNA Technologies. Microscopy experiments were performed in 18-well μ-slide glass bottom chambers (cat #81817) from Ibidi.

1.2 Atomic force microscopy

RNA origami solution was pre-diluted to avoid structure overlap on the mica, if necessary. Subsequently, 20 µL of the solution mixed with 20 µL of imaging buffer (40 mM Tris-Acetate containing 1 mM EDTA and 20 mM Mg^{2+}) were deposited onto a freshly cleaved mica surface (0.95 cm diameter, Science Services GmbH, Munich, Germany) and allowed to adsorb for a minimum of 1 min. The liquid chamber was then filled with 1 mL of imaging buffer. The sample was imaged using Nanowizard 2 high-speed atomic force microscope (Bruker) in either AC fast imaging mode or QI^{TM} mode with a JPK Nanowizard ULTRA Speed using FASTSCAN-D cantilevers (f = 110 kHz, k = $0.25\,N\,m^{-1}$, Bruker Nano Inc., Camarillo, CA, USA).

For characterization, the three RNA origami designs (WT, iSpi and dsOV) underwent IVT as described in Methods, Synthesis of RNA origami in bulk. For the conditions with dye, the IVT was carried out in presence of DFHBI-1T dye at 62.5 µM. RNA origami was then purified using centrifugal filters with a pore size of 100 kDa (Merck Millipore). The filters were pre-wetted with 1x PBS (pH 7.4) containing 20 mM Mg^{2+}. IVT solution was then added to the filter and filled up to 500 µL with the same buffer. After centrifugation for 5 min at 13 000 g, the solution was adjusted up to 50 µL with 1x PBS. The purified solution was then directly deposited on mica for AFM imaging as described above.

1.3 Confocal Microscopy

All images were acquired on a LSM 900 confocal laser scanning microscope from Carl Zeiss AG, Germany equipped with two excitation lasers (488 nm and 640 nm). For the live observation of the RNA origami expression (Fig.2), the 20x air objective was used. Expression of RNA origami nanotubes was imaged using a 63x water

objective. The GUV membrane was labelled with DiD (λ_{ex} = 644 nm, λ_{em} = 665 nm)(Sigma-Aldrich). The RNA origami contained an iSpinach aptamer which binds the fluorophore DFHBI-1T.

1.4 Analysis of RNA origami expression in GUV

To quantify the fluorescence intensity within GUVs where transcription was triggered with Mg^{2+} or rNTPs (as shown in Fig. 2g,h, 5b, S6, S8, and S23), GUVs were selected using the DiD channel (λ_{ex} = 640 nm, λ_{em} = 665 nm). The fluorescence intensity was then analyzed in the DFHBI-1T channel (λ_{ex} = 488 nm, λ_{em} = 505 nm). A region of interest (ROI) for each GUV was delineated with a circular mask on an 8-bit grayscale image using Image J (1.53v). To analyse the transcription trigger over time, the same GUV timeline image (as shown in Supplementary movie 1, 2, 3, 4) was used at 2 min interval up to 3 h to 4 h. The mean intensity values obtained for each GUV were obtained by using Measure function and presented as fluorescence intensity across the samples.

1.5 RNA origami bundling

RNA was transcribed *in vitro* for 2 h. Subsequently the samples were heated to 75 °C for 1 min and then cooled down to 25 °C with a temperature gradient of $-0.5\,°C\,min^{-1}$ in a thermocycler (BioRad). For bundling, 1 µL of annealed RNA origami was mixed with 20 µL of imaging buffer (40 mM Tris-Acetate containing 1 mM EDTA and 20 mM Mg $^{2+}$) and deposited onto a freshly cleaved mica to adsorb for 5 min. The AFM images were processed using Gwyddion. Five width measurements, equally distributed along the measured object, were performed and the averaged widths were reported in Fig. 3i.

2 Supplementary Figures

2.1 Expression of RNA origami inside GUVs (Figs. S1-S8)

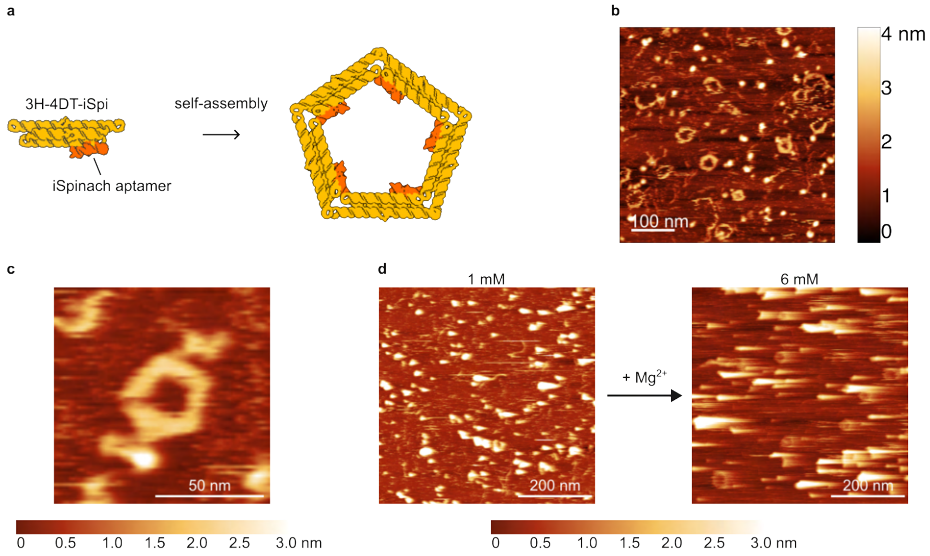

Fig. S1 Verification of RNA origami production. **a)** Schematic representation of the 3H-4DT-iSpi RNA origami design. **b)** AFM image of the 3H-4DT-iSpi RNA origami folded co-transcriptionally in a buffer containing 6 mM Mg^{2+}. The 3H-4DT-iSpi RNA origami design is taken from Geary et al. [1]. **c-d)** The height images corresponding to the micrographs in Figure 2c-d, respectively.

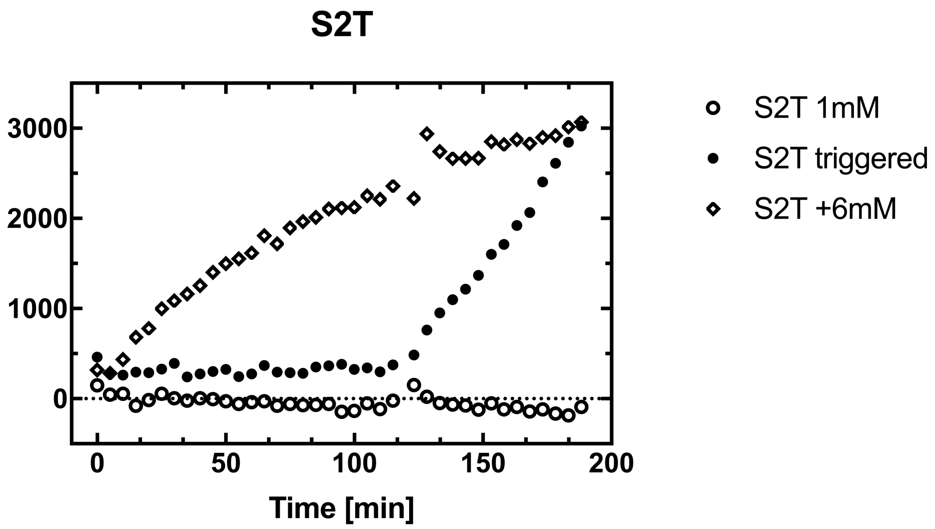

Fig. S2 S2T RNA origami production upon Mg^{2+} addition. Mean spectrophotometer readout of DFHBI-1T fluorescence (λ_{ex} = 488 nm) is plotted over time (n = 3 wells per condition). At 1 mM $Mg(OAc)_2$ (empty circles), the fluorescence remains constant, indicating that no RNA origami is produced. However, addition of 5 mM $Mg(OAc)_2$ to reach a final concentration of 6 mM (here at t = 120 min) leads to an immediate increase in fluorescence, indicative of RNA origami production (filled circles). This increase is also seen in a positive control where the initial solution contains 6 mM $Mg(OAc)_2$ throughout the entire measurement (empty diamonds). This confirms that RNA origami production can be triggered by the addition of Mg^{2+}. The S2T RNA origami design is taken from Geary et al [1].

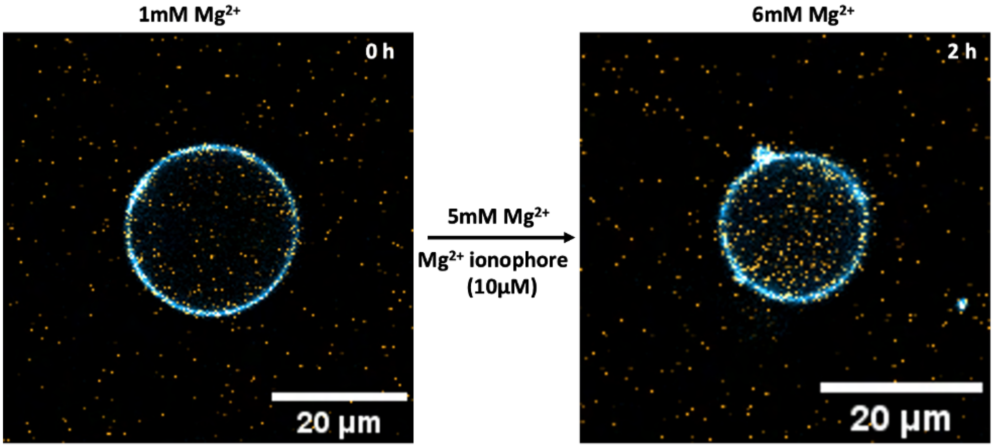

Fig. S3 Expression of the 3H-4DT-iSpi RNA origami in GUVs triggered by externally added Mg^{2+}, transported into the GUVs by using ionophores. Left: GUV containing 1 mM Mg^{2+} and all other transcriptional components along with rNTPs. Right: Transcription of 3H-4DT-iSpi RNA origami was triggered by addition of 5 mM Mg^{2+} and 10 µM Mg^{2+} ionophore. Image taken after 2 h incubation at 37 °C.

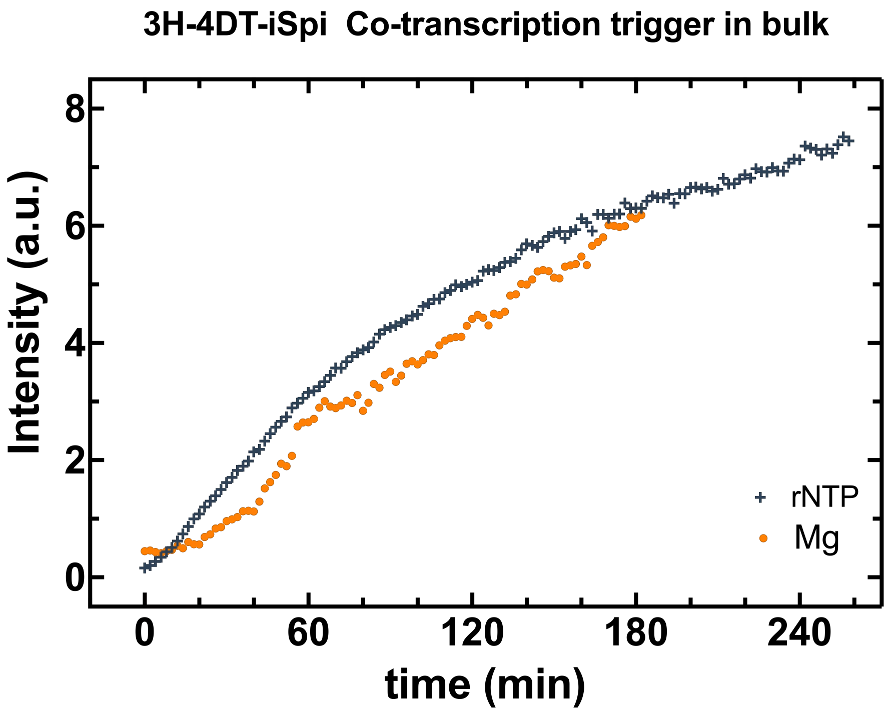

Fig. S4 RNA origami production in bulk measured with confocal microscopy. Mean fluorescence intensity of 3H-4DT-iSpi RNA origami was monitored at 37 °C. Transcription was triggered by the addition of 5 mM Mg^{2+} (orange circles) or with 4 mM rNTPs (gray crosses) for 3 h or 4 h, respectively.

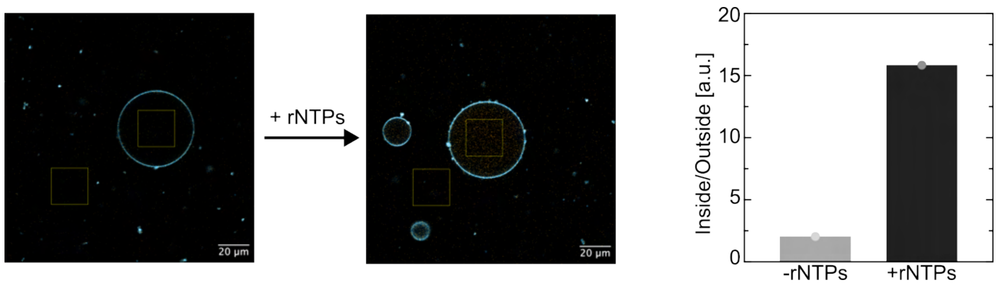

Fig. S5 Quantification of the increase in fluorescence after addition of rNTPs shown in Fig. 2f. The mean intensity inside the GUV (highlighted by the yellow box) compared to the exterior region in the same image, both in the absence and presence of rNTPs. A 7-fold higher mean intensity was observed upon the addition of rNTPs.

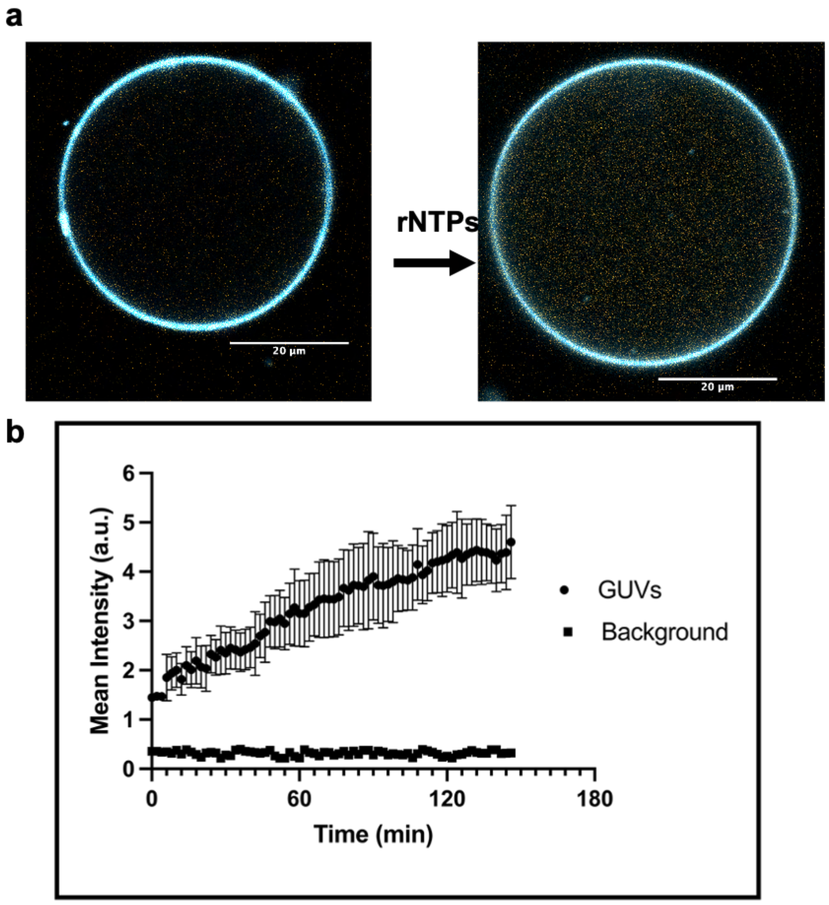

Fig. S6 S2T RNA origami transcription and folding inside GUVs triggered by rNTP addition. **a)** Confocal images showing S2T RNA origami expression initiated by the external addition of 4 mM rNTPs. GUVs were incubated for 2 h before the left image was taken; the right image was taken 2 hours after transcription was initiated. rNTP fuelling was achieved by the presence of α-hemolysin in the GUV membrane. **b)** S2T RNA origami transcription inside of GUVs triggered by addition of rNTPs plotted over the first 2.5 h of expression (mean ± s.d, $n = 10$ GUVs). The data was extracted from confocal fluorescence timelapse recordings (Supplementary Movie 3). Background fluorescence outside of the GUVs is plotted as black squares.

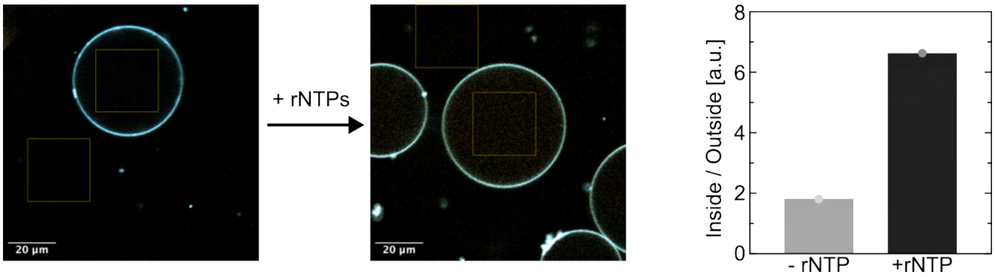

Fig. S7 Quantification of the increase in fluorescence after addition of rNTPs shown in Figure S6a. The mean intensity inside the GUV (highlighted by the yellow box) compared to the exterior region in the same image, both in the absence and presence of rNTPs. A 3-fold higher mean intensity was observed upon the addition of rNTPs.

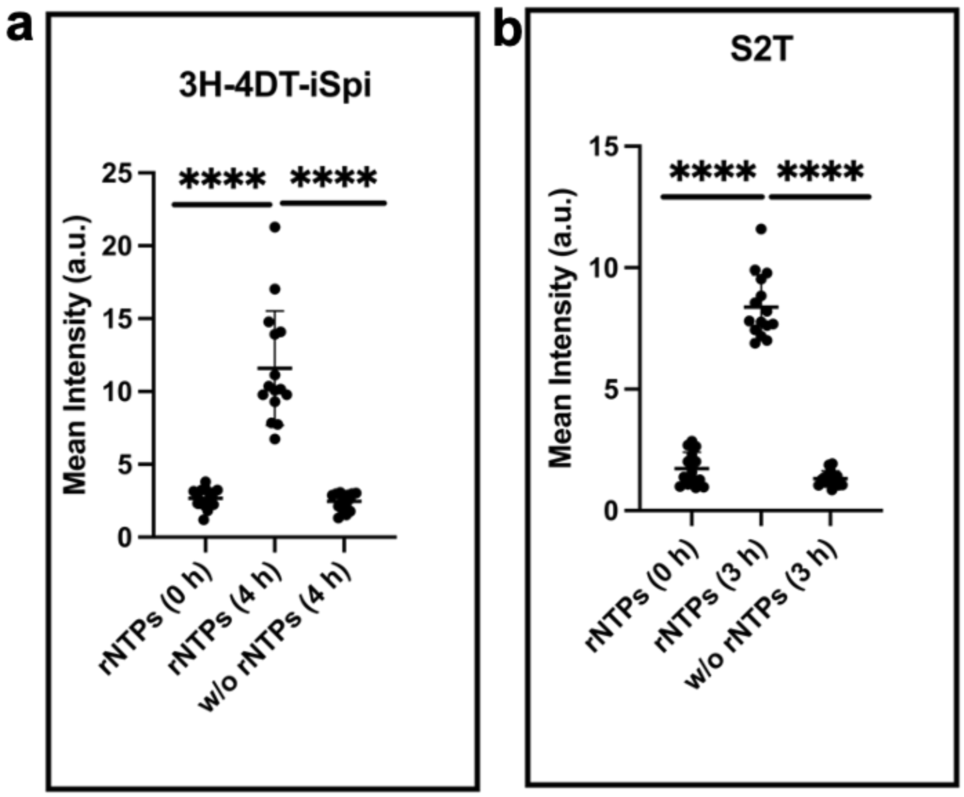

Fig. S8 RNA origami transcription in GUVs triggered by rNTP addition and measured by confocal microscopy. Transcription was carried out at 37 °C. GUVs (n = 15) were initially randomly chosen in the membrane dye channel (DiD), followed by a comparison of their fluorescence intensity in the iSpinach channel. **a)** The mean fluorescence intensity of 3H-4DT-iSpi RNA origami increased significantly 4 h after the addition of rNTPs, compared to the intensity before or without the addition of rNTPs. **b)** Mean fluorescence intensity of S2T RNA origami increased significantly 3 h after the addition of rNTPs, compared to the intensity before or without the addition of rNTPs. The mean and standard deviation are represented in black lines. Parametric, unpaired t tests with Welch's correction were performed. Two-tailed P values are marked with **** (p = 3.3e−7, 2.7e−7, 7.6e−14, 1.5e−12 , from left to right).

2.2 Construction of RNA origami nanotubes (Figs. S9-S19)

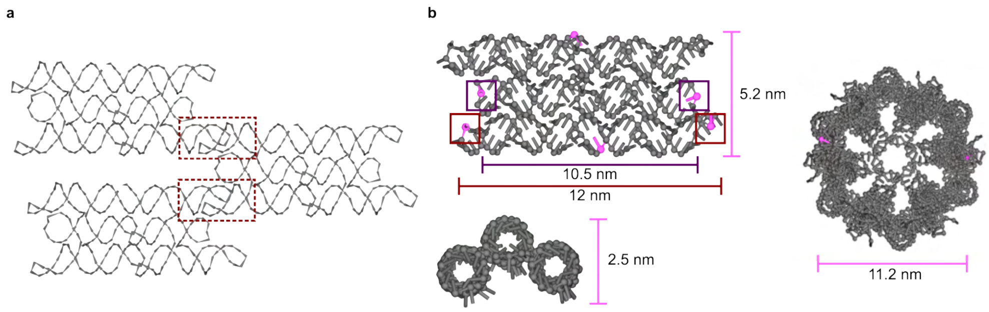

Fig. S9 *In silico* design of the wildtype (WT) RNA origami tiles for nanotube formation. **a)** Schematic of intertile interactions indicating the direction of the external kissing loops (dotted red box). To minimize the number of components, we employed 180° kissing loops for homotypic tile-tile interactions. These external kissing loops allow for more tile-tile cohesion points per strand, which are necessary for strengthening the nanotubes, than traditional sticky-end hybridization methods. Additionally, this approach allows us to compose each tile from a single strand rather than the conventional five strands per tile used in double-crossover (DX) tile motifs known from DNA nanotechnology [2–4]. Furthermore, unlike traditional multi-strand RNA/DNA nanotube designs where intrinsic curvature is introduced at the tile-tile interaction level using offsets in the helix axes [3], we introduced curvature directly in individual tiles with dovetail junctions (−3 dovetail) [1, 5]. This produced the desired radial curvature of 120° for a three-tile nanotube where each tile is connected corner-to-corner by 180° kissing loops. Given that the 11 base-per-turn characteristic of the RNA helix does not enable symmetrical tile designs, we optimized the loop lengths by visual inspection, and generated the sequence using Revolvr [1, 6]. **b)** Dimensions of the WT tile and the resulting RNA origami nanotube extracted from RNAbuild[1] of the *in silico* design. The nucleotides used as reference points for measurement are highlighted in pink.

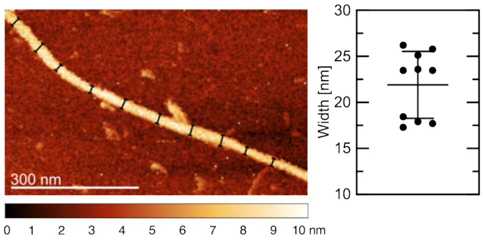

Fig. S10 AFM micrographs of cotranscriptionally folded RNA origami nanotubes. High magnification AFM micrographs of a WT RNA origami nanotube. Ten width measurements from one exemplary nanotube were performed along the black lines and shown in the plot on the right ($n = 10$). The measurements yielded dimensions of 21.91 ± 3.632 nm. The mean and standard deviation are represented in black lines.

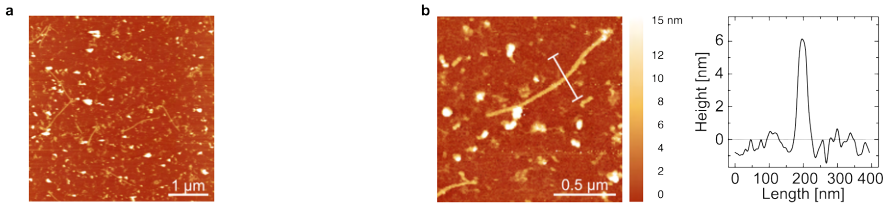

Fig. S11 Stability of RNA nanotubes. **a)** AFM micrograph of cotranscriptionally folded RNA origami nanotubes after incubation at 50 °C for 1 h. **b)** A zoom in showing the height profile of the RNA nanotube at the white line. The RNA nanotube was intact and at the correct height of 6 nm. In contrast, DNA nanotubes have been shown to completely disintegrated at 46 °C after 30 min [7]. The AFM micrographs acquired in this paper were done at 50-100 pN on mica substrate and imaging was done between 20-90 min. The RNA nanotubes remained unopened during the imaging process.

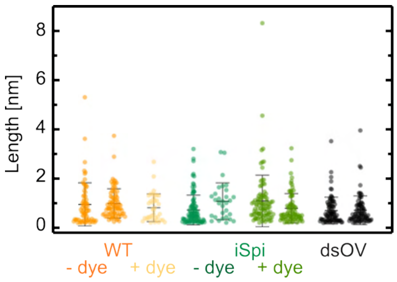

Fig. S12 Contour length distribution of RNA origami nanotubes extracted from AFM images. Wild-type: WT; iSpinach aptamer on the 3' end: iSpi; and double-stranded overhang on the 3' end: dsOV. Mean lengths of WT, iSpi and dsOV (from left to right, all replicates pooled) are: 970.9 ± 735.1 nm and 814.8 ± 564.1 nm; 802.1 ± 657.0 nm and 952.4 ± 883.3 nm; and 706.1 ± 563.8 nm, respectively. Each column represents one replicate. All conditions have 2 replicates except WT+dye. $n = 152$ (69 and 83), 38, 133 (103 and 30), 175 (95 and 80), 159 (80 and 79) for WT, WT+dye, iSpi, iSpi+dye and dsOV, respectively. Mean and standard deviation are presented in black lines. The spread of the contour length is indicative of possible frequent assembly defects.

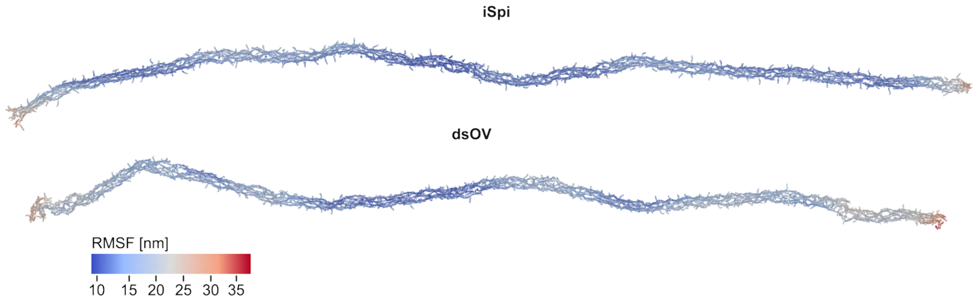

Fig. S13 Coarse-grained molecular dynamics simulation of a 300-tile assembly (top: iSpi, bottom: dsOV). The centroid structure from one of the simulation runs is shown. Each nucleotide is color-coded according to its root-mean-square fluctuations (RMSF).

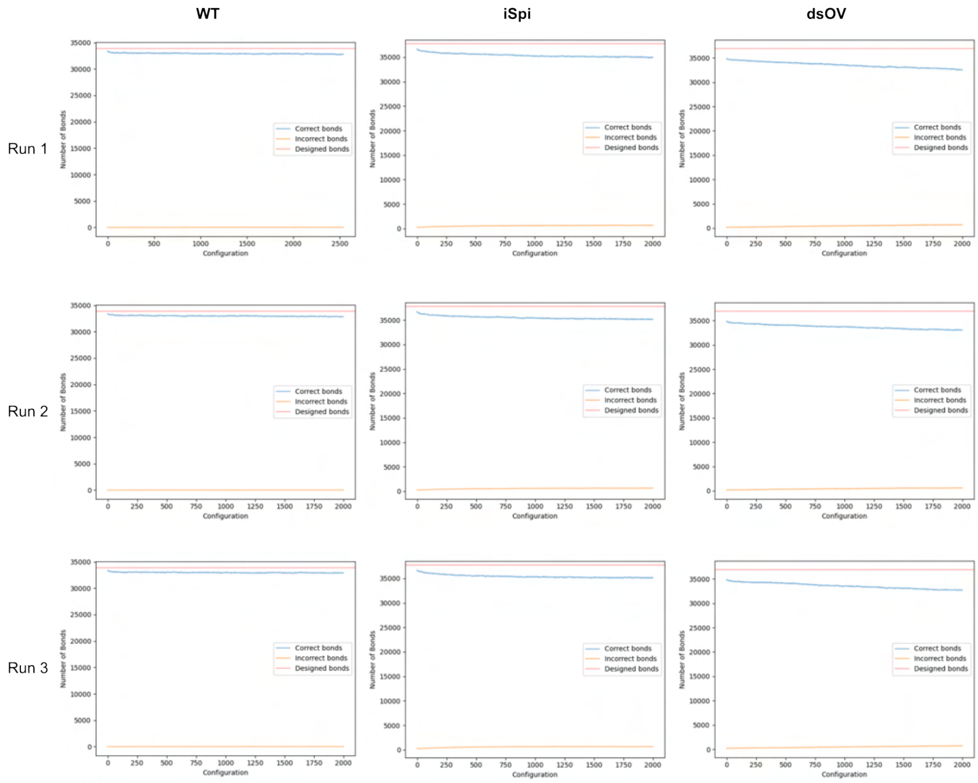

Fig. S14 Bond occupancy compared with the target design of 300-tile RNA origami nanotubes over the course of equilibrium oxRNA simulations (1e9 steps, dt = 0.003). The graphs are organized by column (designs) and row (simulation run replicate). The number of correct bonds experienced a decrease at the beginning of the simulation, then remained at a constant level for WT and iSpi designs. In contrast, dsOV design exhibited a constant decrease in the number of correct bonds throughout the simulation.

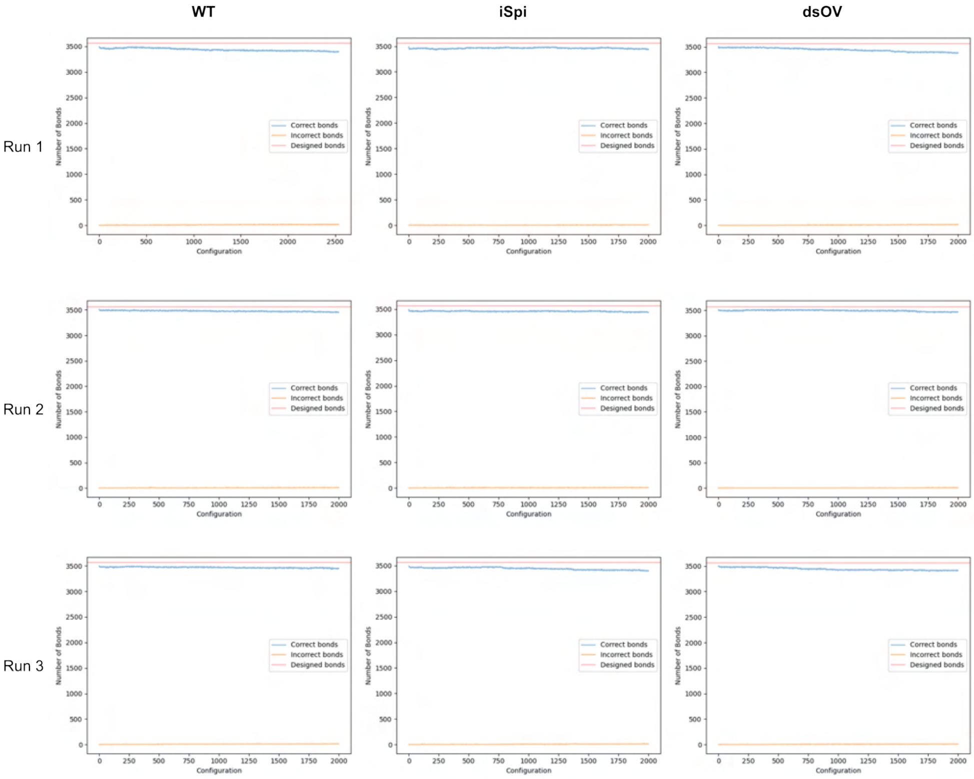

Fig. S15 Bond occupancy in intertile kissing loops of 300-tile RNA origami nanotubes over the course of equilibrium oxRNA simulations (1e9 steps, $dt = 0.003$). The graphs are organized by column (designs) and row (simulation run replicate). Throughout the simulation, the number of correct bonds is maintained for all three designs. Note that the overall bond occupancy of the dsOV design decreases consistently (Figure S14), while the inter-tile bonds remain stable and correct. This indicates that bond breakage primarily occurs within the internal bonds of the tile.

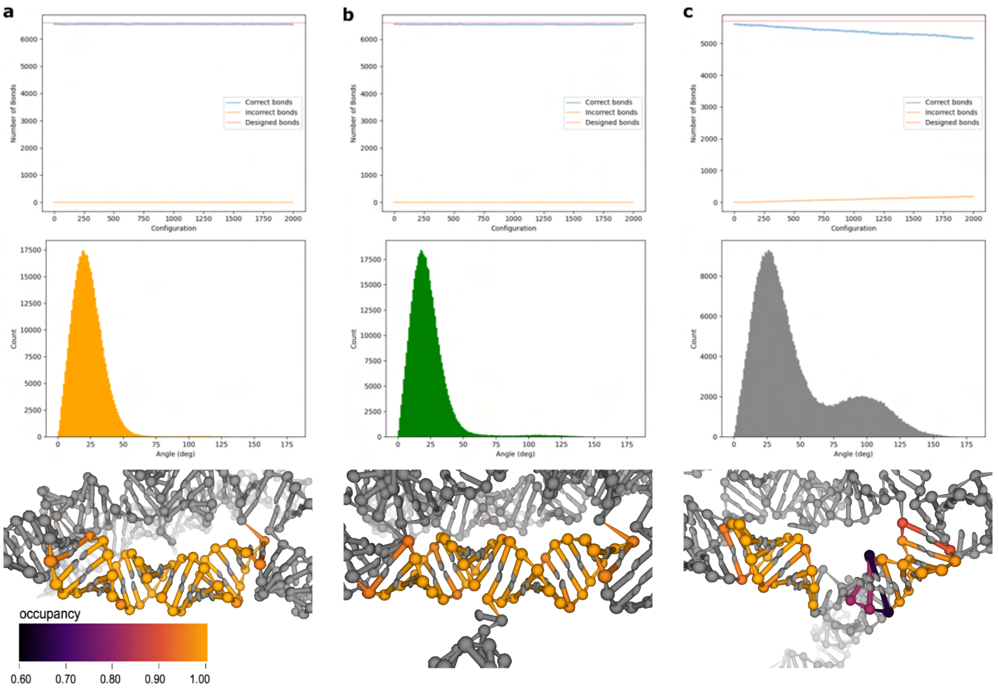

Fig. S16 Base-pairing patterns (top, bottom) and angle across nick (middle) from oxRNA simulations of the 1 µm filament. Each column corresponds to a tile design: WT (**a**), iSpi (**b**), and dsOV (**c**). The data is for all 300 tiles in the helix (base-pairing) or for the middle 240 tiles (angles). Data from just one replicate shown here, however the pattern of both bond occupancy and angle distribution are similar for all three replicates.

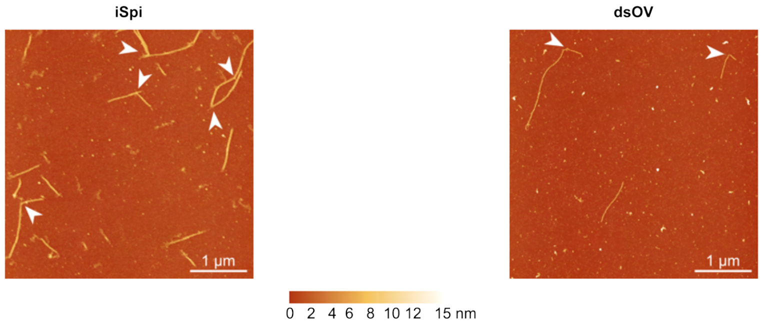

Fig. S17 AFM micrographs of RNA origami nanotubes displaying kinks and branching (highlighted by white arrowheads) in the structure. The nanotubes are made from iSpi tiles (height channel, left) and from dsOV tiles (height channel, right).

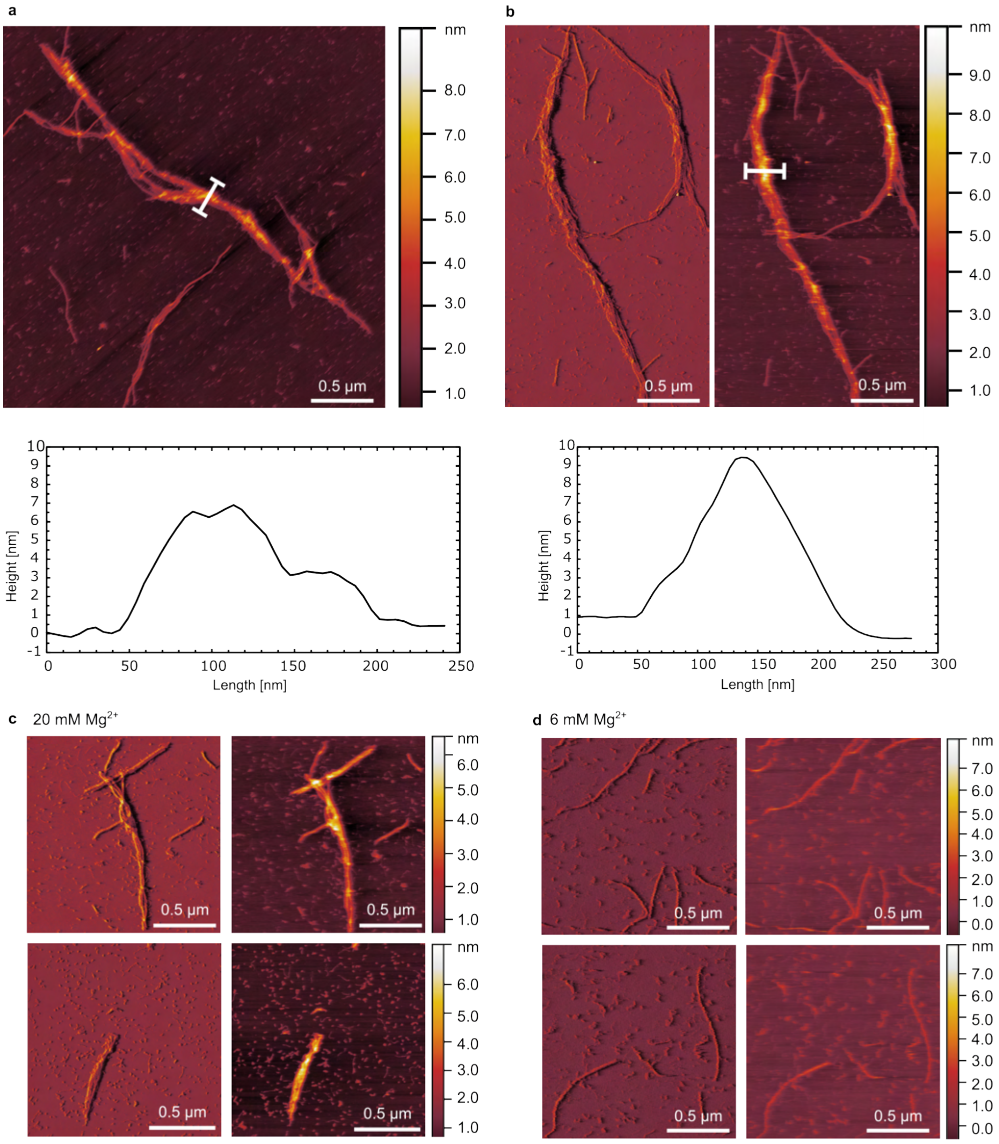

Fig. S18 Examples of bundling of annealed RNA origami nanotubes (dsOV design) upon addition of Mg^{2+} (20 mM) as a crosslinker. **a-b)** AFM micrographs of bundled RNA origami nanotubes. **a)** shows the height channel corresponding to the AFM image in Fig. 3h. **b)** shows another RNA origami nanotube bundle. The height profiles corresponds to the height measurement along the white lines. **c-d)** AFM micrographs of RNA origami nanotubes at bundles at 20 mM Mg^{2+} **(c)** and 6 mM Mg^{2+} **(d)**. The error signal and height channel is shown on the left and right side, respectively, for **b-d**.

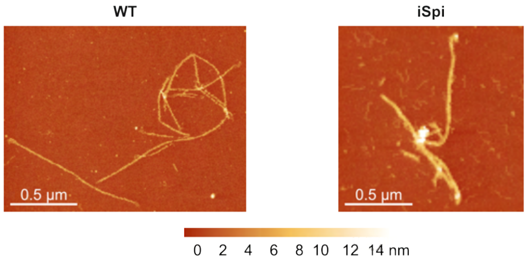

Fig. S19 AFM micrographs of thermally annealed RNA origami nanotubes of the WT and iSpi design in 20 mM Mg^{2+} after $>$ 45 min incubation.

2.3 Formation of RNA origami rings (Figs. S20-S22)

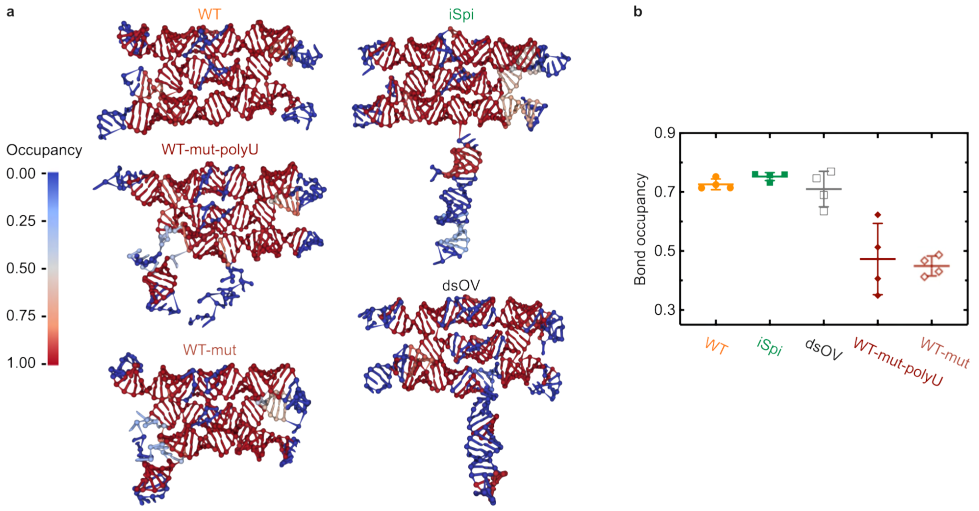

Fig. S20 Bond occupancy of single-tile simulations. **(a)** OxRNA molecular dynamics simulations of the RNA origami tile designs. The centroid structure of the concatenated trajectory from 4 simulation runs is shown. Each nucleotide is color-coded according to its bond occupancy compared with the target design throughout the simulation. **(b)** Bond occupancy of the hairpin highlighted in Fig. 4 across designs (mean ± s.d, $n = 4$ simulation runs). Note that the 4-nucleotide tetraloop at the ends of the hairpin are "unpaired" in the secondary structure, thus the maximum occupancy value around 0.75. The data from Fig. 4g is replotted for comparison in **(b)**.

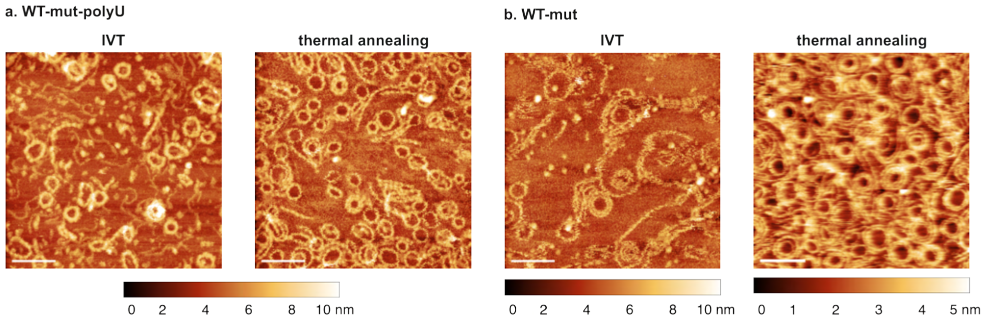

Fig. S21 AFM micrographs of ring-forming RNA origami assembled from WT-mut-polyU tiles **(a)** and WT-mut tiles **(b)**. The RNA origami was formed by co-transcriptional folding (left, IVT) or by thermal annealing (right). The height image of the thermally annealed RNA origami ring of the WT-mut design corresponds to the micrograph in Figure 4. Scale bars: 100 nm.

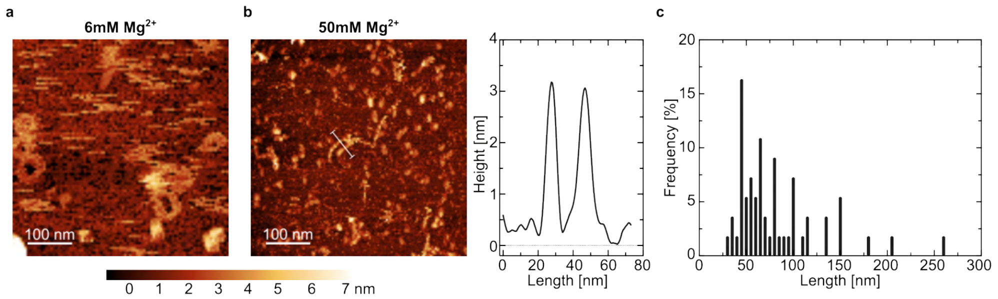

Fig. S22 Phenotype transition from nanorings to filaments in response to Mg^{2+} concentration of WT-mut design. AFM micrographs of RNA nanostructures produced after 2 h IVT at 6 mM Mg^{2+} (**a**) and 50 mM Mg^{2+} (**b**). The height profile of the RNA filaments at the white line is shown in (**b**). (**c**) Histogram of the length of RNA filaments produced at 50 mM Mg^{2+} ($n = 55$ filaments).

2.4 Expression of an RNA origami cytoskeleton in synthetic cells (Figs. S23-S28)

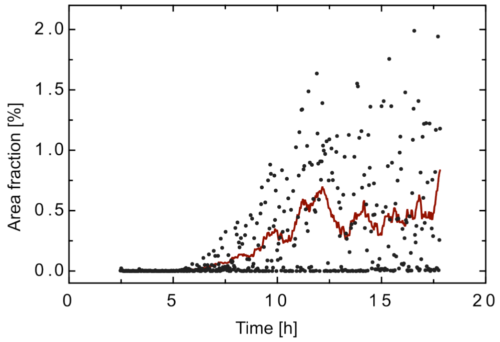

Fig. S23 Tracking of RNA origami nanotube growth inside a single GUV after rNTP triggering. The area fraction is plotted over time (2 min interval). Fluctuations of the area fraction are inevitable due to the constant Brownian diffusion of the RNA origami nanotubes in and out of the focus plane. The red line is drawn to guide the eye, obtained by curve smoothing in Prism (Version 10.2.3) using 10 neighbors on each side with 0th order smoothing polynomial. The area fraction was extracted from a confocal time series using similar method as in Fig. 5c. The GUV detection and measurements were done automatically using a custom-written code (see Code availability). The data was then filtered using a custom-written Python code (see Code availability).

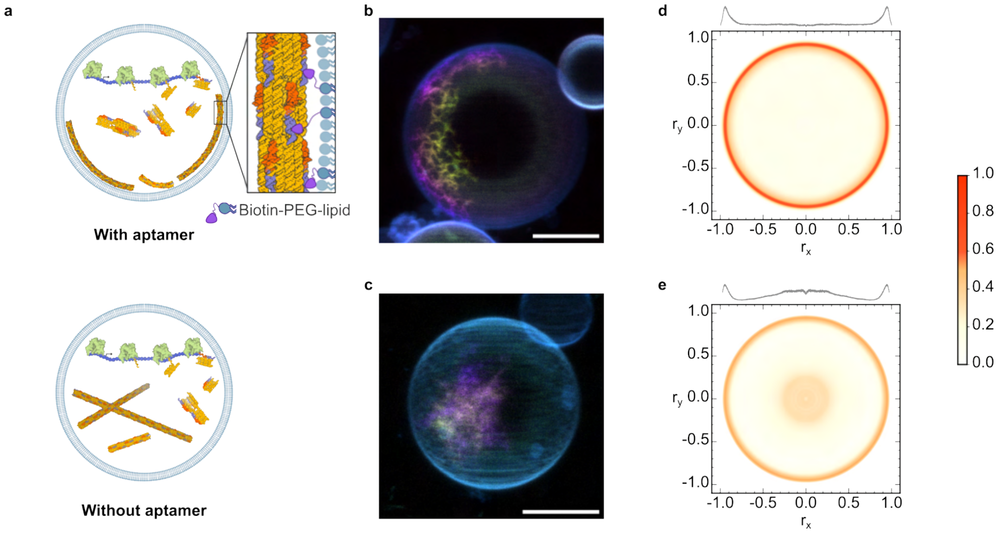

Fig. S24 Functionalized RNA origami nanotubes for membrane binding. **a)** Schematic representation of the biotin-aptamer containing RNA origami nanotubes for binding to biotinylated GUV membranes. **b-c)** Confocal z-stack projections of biotin-aptamer containing **(b)** and aptamer-free **(c)** iSpi RNA origami nanotubes inside a GUV. **(b)** shows the z-projection of the image presented in Fig. 5d. The membrane is labelled with DiD ($\lambda_{ex} = 640\,\mathrm{nm}$, blue). The RNA origami z-projection is colour coded. **d-e)** Radial fluorescence distribution of the RNA origami nanotubes with **(d)** ($n = 78$) and without biotin aptamer **(e)** ($n = 87$) inside the GUVs. Membrane binding is indicated by a shift in the center of mass of the radial RNA fluorescence signal towards the biotinylated membrane. Scale bars: 10 µm.

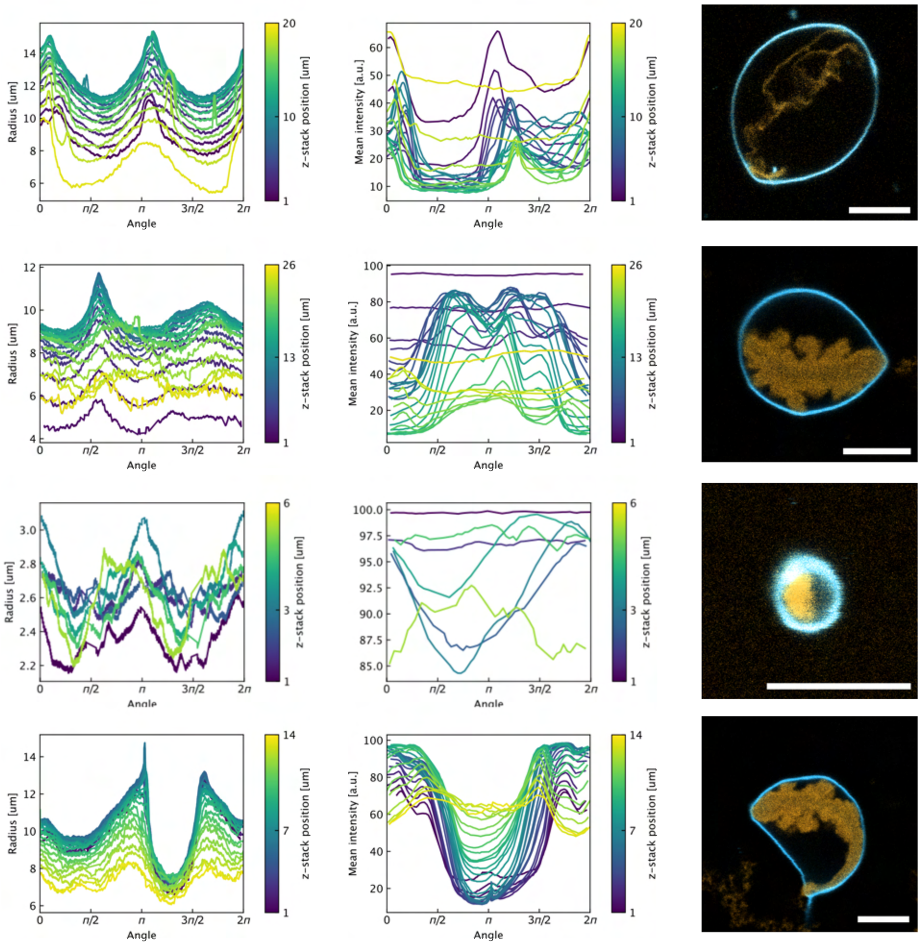

Fig. S25 Analysis of confocal z-stacks of multiple deformed GUVs ($n = 4$). Deformation is caused by the production of RNA origami nanotubes with biotin aptamers inside the GUV. **Column 1-2:** Angular distribution of the GUV radii and RNA fluorescence signal, respectively, over the perimeter of the GUV. The analysis was done over all z-stacks. **Column 3:** RNA nanotube-expressing GUVs were incubated for 6 h to 8 h prior to imaging. The membrane is labelled with DiD ($\lambda_{ex} = 640$ nm) shown in blue and RNA nanotube ($\lambda_{ex} = 488$ nm) shown in orange. Scale bars: 10 µm. Peaks represent the maximum distance from the center, corresponding to membrane deformations caused by the RNA nanotubes. For example, lemon-shaped deformed GUVs show two peaks (angular distance between the two peaks $= \pi$), whereas one-sided elongated GUVs exhibit a single peak, and non-deformed, round GUVs display no peaks (see Fig S28). Deformation is more pronounced in the middle of the Z-stack compared to higher and lower Z-stack positions.

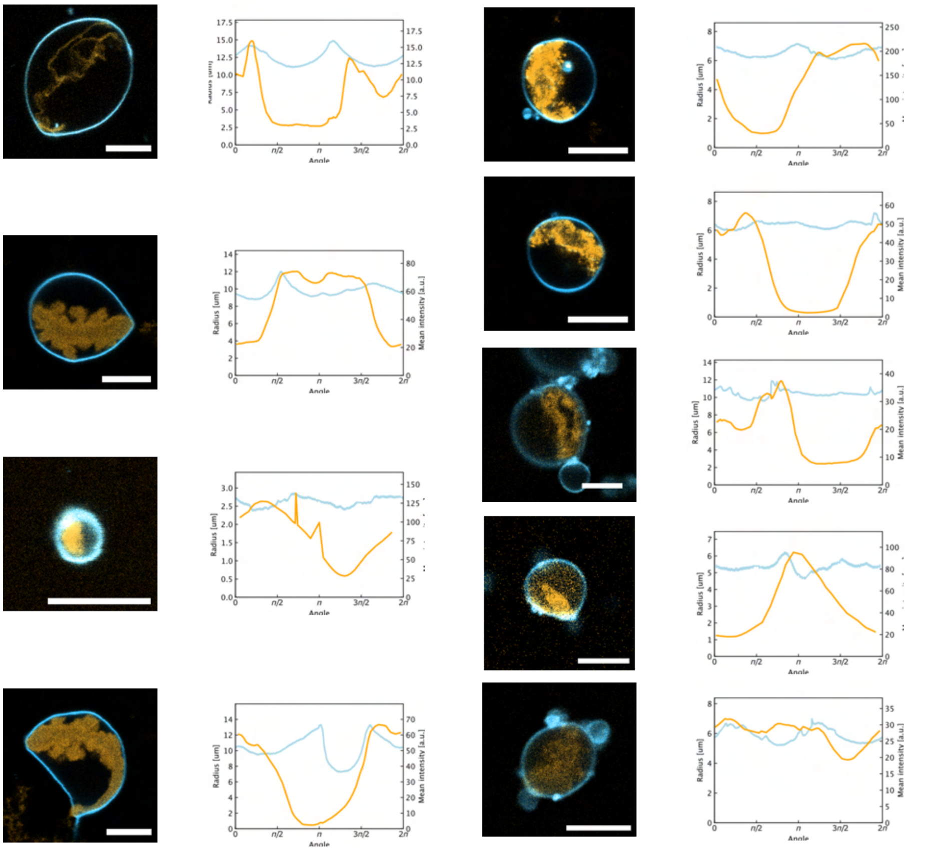

Fig. S26 Deformation of GUVs containing RNA origami nanotubes with biotin aptamers. Confocal images of multiple deformed GUVs ($n = 9$) and corresponding angular distribution of GUV radii (blue) and RNA intensities (orange) over the GUV perimeter. GUVs were incubated with all transcription components, including rNTPs, at 37 °C for 6 h to 8 h prior to imaging. The angular distributions are plotted from a 2D plane of a single GUV image. Scale bars: 10 µm.

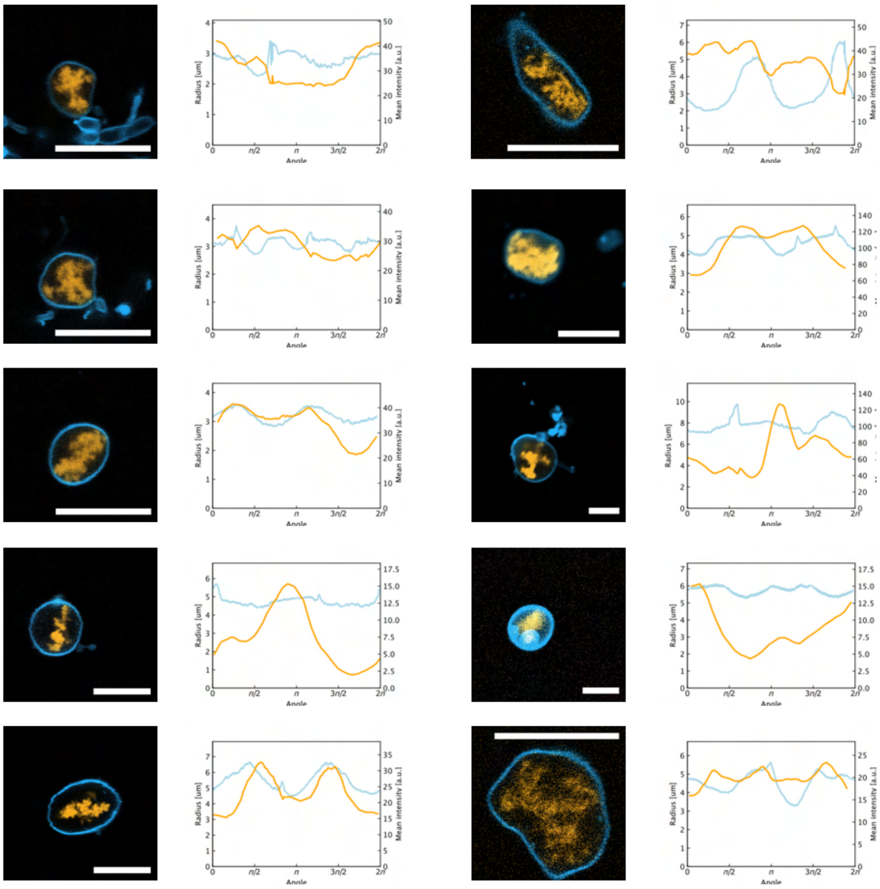

Fig. S27 Deformation of GUVs containing RNA origami nanotubes without biotin aptamers. Confocal images of multiple deformed GUVs (n = 10) and corresponding angular distribution of GUV radii (blue) and RNA intensities (orange) over the GUV perimeter. GUVs were incubated with all transcription components, including rNTPs, at 37 °C for 6 h to 8 h prior to imaging. The angular distributions are plotted from a 2D plane of a single GUV image. Scale bars: 10 µm.

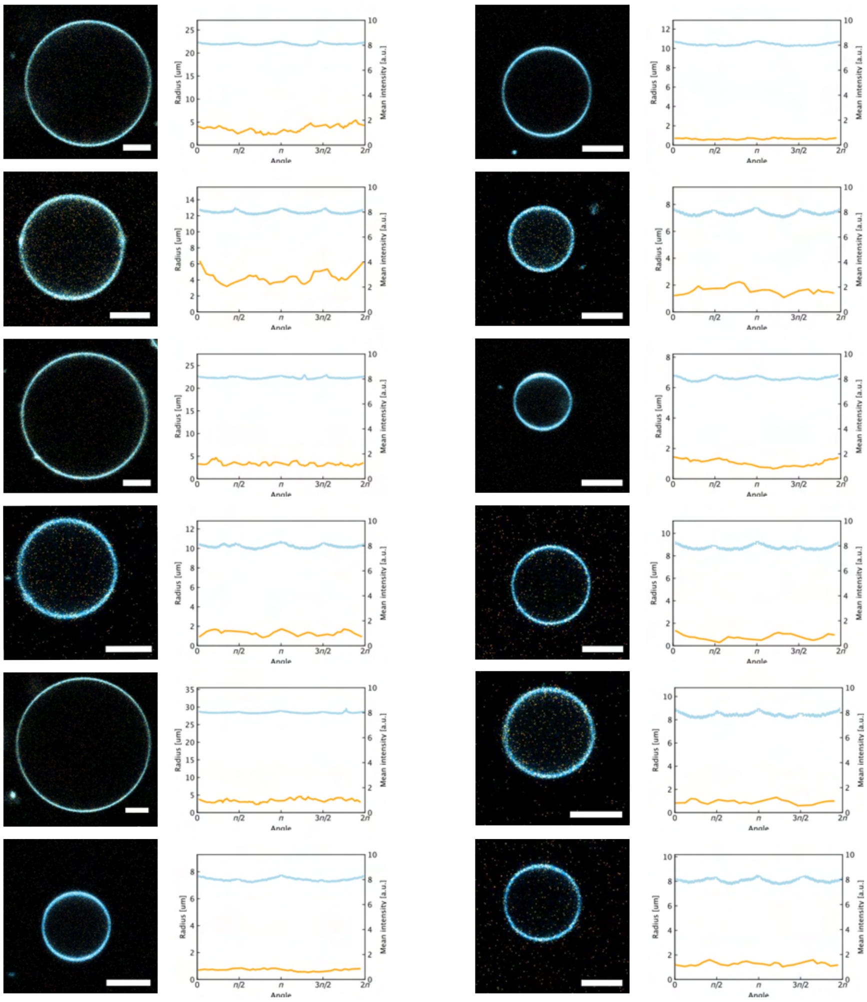

Fig. S28 Undeformed GUVs in the absence of RNA origami nanotube formation within the GUV compartments. Confocal images of multiple undeformed GUVs ($n = 12$) and corresponding angular distribution of GUV radii (blue) and RNA intensities (orange) over the GUV perimeter. GUVs were incubated with all transcription components, excluding rNTPs. The angular distributions are plotted from a 2D plane of a single GUV image. Scale bars: 10 µm. No peaks were observed in the round GUVs, indicating the absence of deformation.

2.5 RNA origami blueprints

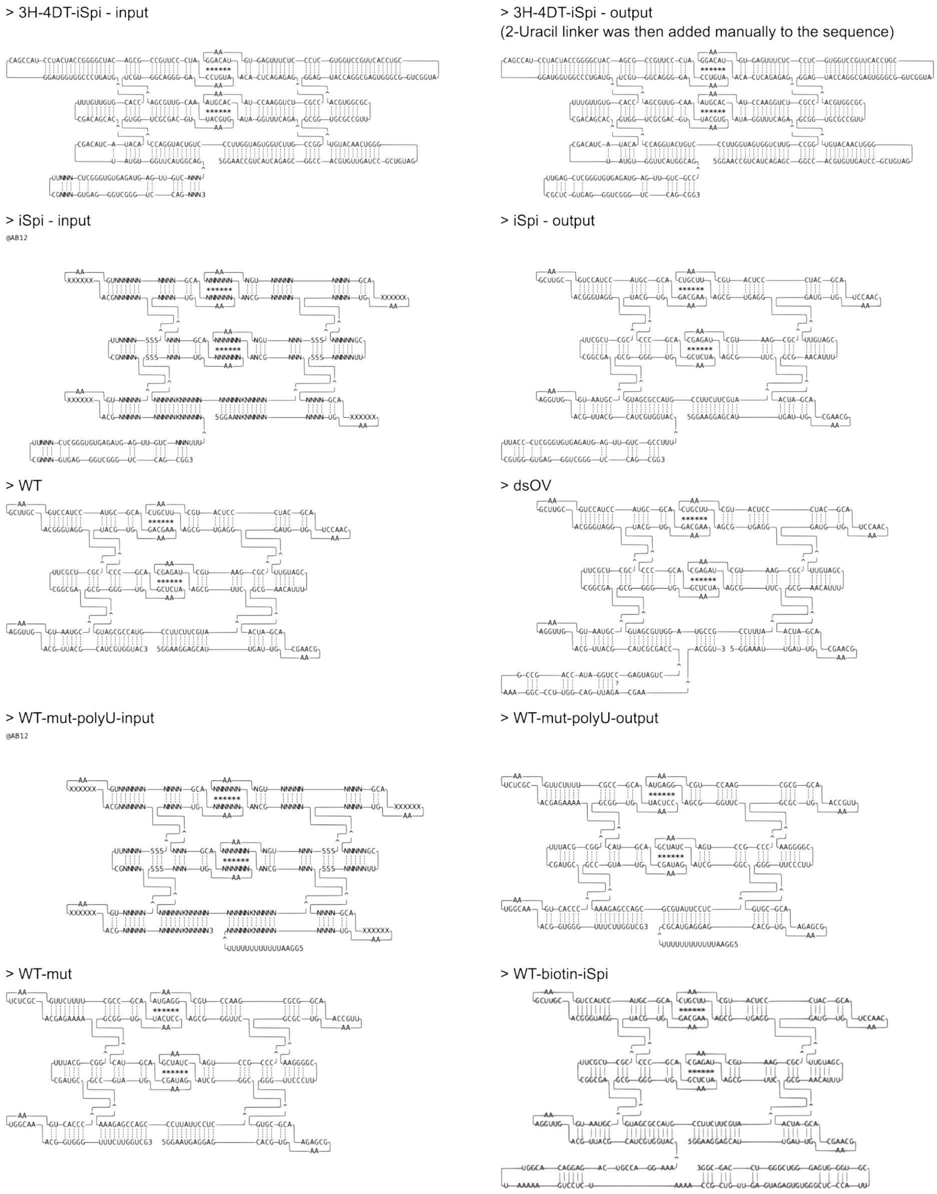

Fig. S29 The blueprints of all RNA origami designs used as input for the RNA origami design software ROAD. For the designs where the blueprint was used as an input for Revolvr[1], the input and output blueprints were specified. All blueprint text files are uploaded on GitHub (see Code Availability).

3 Supplementary Data 1: Sequences

All the DNA templates and primers sequences are provided in an excel sheet appended as Supplementary Data 1.

4 Supplementary Notes

4.1 Persistence length analysis

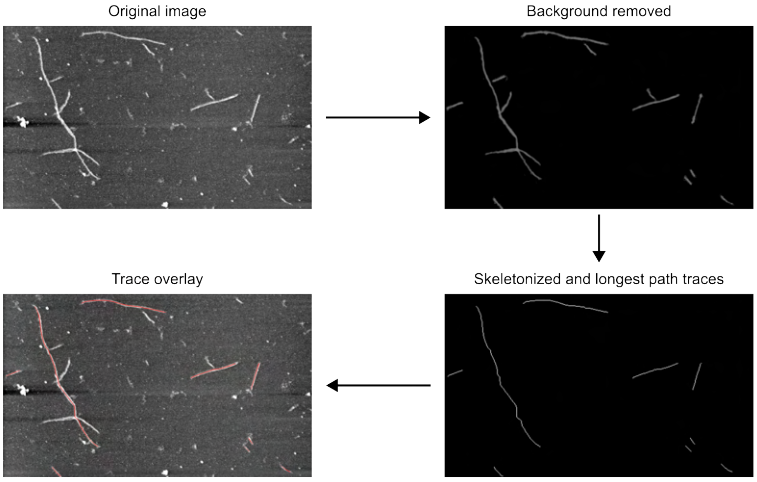

Fig. S30 Image analysis workflow for detection of RNA nanotubes. Example of an image of RNA nanotubes from iSpi design. The AFM micrograph is first exported as a TIFF image. The background was then removed with the help of Selection Tools in Adobe Photoshop 2024. The background-removed images were then skeletonized and the longest path for each nanotube was traced (See Methods, Persistence length analysis). The traces were then exported as XY coordinates and used for contour and persistence length analysis.

Due to the large distribution in the contour lengths of the nanotubes observed in AFM imaging, we used the mean squared end-to-end distance (MSED) method to determine persistence lengths (Figure S31).

$$\langle R^2 \rangle_{3D} = 2sPL\left(1 - \frac{sP}{L}(1 - e^{-L/sP})\right) \tag{1}$$

Where $\langle R^2 \rangle$ is the mean squared end-to-end distance, P is the persistence length, and L is the contour length. s is a surface parameter which is equal to 2 for a 2D surface, and 1 for 3D measurements. Since the AFM imaging is done in solution, we used $s = 1$ [8, 9].

We pooled all imaged nanotubes from 2 independent experiments, with the exception of WT+DFHBI which was only tested once (see Figure S12).

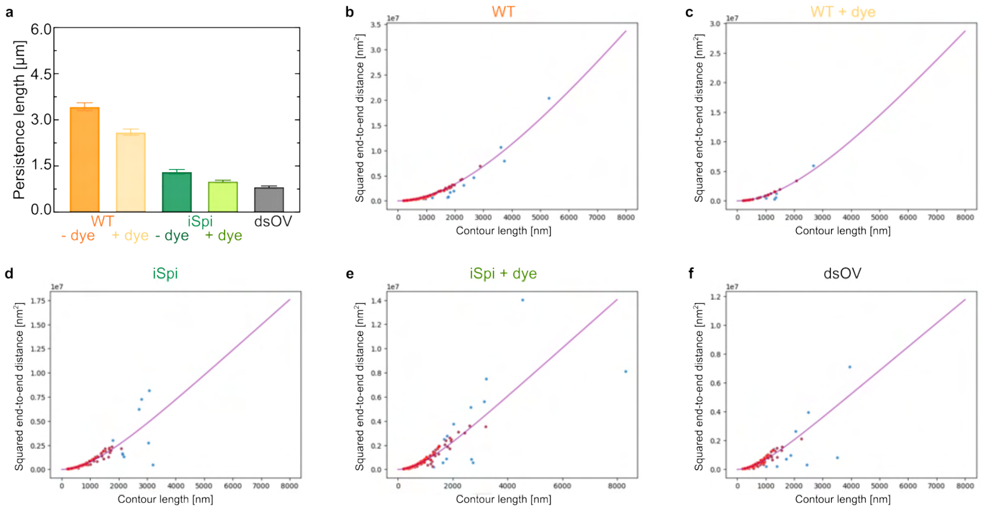

Fig. S31 Fitting persistence length to graphs of contour length vs squared end-to-end distance. (a) Fitted persistence lengths for all tile designs (WT and iSpi with and without DFHBI dye) (fitted value ± standard error of fit) (b) WT tile ($n = 141$). (c) WT tile with DFHBI dye ($n = 33$). (d) iSpi tile ($n = 124$). (e) iSpi tile with DFHBI dye ($n = 162$). (f) dsOV tile ($n = 149$). Length measurement was performed using all nanotubes (blue), while the persistence length fit was done using only those points within 1 standard deviation of an initial fit (red).

For the oxRNA simulations of nanotubes, the length is limited within a small range (since all nanotubes contain exactly 300 tiles). Therefore the $\langle R^2 \rangle$ measurements were generated by measuring the distance between slices of the nanotube, where the midpoint of each slice was defined as the center of mass of the three tiles that made up each layer in the nanotube. Fitting to all pairwise distance, each trajectory frame yields one persistence length and the standard error of the fit. These persistence lengths were then averaged and the standard errors were propagated and reported in Fig. 3g.

Note that due to experimental limitation of AFM imaging (resolution and size limit), bending of correctly assembled nanotubes cannot be distinguished from bending caused by assembly defects and regular branching. The analysis takes into account the longest path of any given nanotube regardless of the bending type. The calculated persistence length will therefore report stiffness as a result of not only the material properties but also the assembly success and branching frequency.

The persistence lengths derived from the analysis (Fig. 3f,g, Fig. S31) suggest that both steric hindrance during assembly and tile stability in assembly affect the stiffness of the nanotubes.

If an overhang was disrupting nanotube assembly for purely steric reasons, one would expect the iSpi design to show lower persistence length than the dsOV due to the larger size of the additional structure. However, we do not see this effect, suggesting that the manner of attachment – a ssRNA overhang for the iSpi versus the more rigid 3-way junction for the dsOV – has a significant impact on the ability of the tiles to assemble and the stiffness of the resulting assembly. This hypothesis is

further supported by the instability of the dsOV design in oxRNA simulations, where it showed continuous loss of intra-tile bonds, specifically in the helix contain the overhang, over the course of the simulations at a much higher rate than iSpi or WT (Fig. S14, S15, S16). This increases the flexibility of the branched helix in the dsOV tiles compared with the other two designs (Fig. S16). The steric hindrance hypothesis is supported by the difference between the stiffness of the nanotubes containing overhangs to the WT design. The fact that the nanotubes often exhibit kinks and branching rather than smooth contours in AFM for iSpi and dsOV designs (Fig. S17) also points towards the fact that the primary source of nanotube flexibility are broken bonds, transcription errors, or incorrect incorporation of tiles during assembly. This is consistent with the higher persistence lengths measured in simulations, where the nanotubes were first forced to be perfectly formed, as opposed to the stochastic and error-prone polymerization process in experiments.

4.2 Solution osmolarity

The deformed GUVs were pre-encapsulated with DNA templates and T7 polymerase (see Methods). The DNA template solution was in water with a concentration of 4-8 ng µL^{-1} which is equivalent to 20-40 nM (the DNA template is 220-250 kDa). The T7 polymerase is stored in 50 mM Tris-HCl (pH 8.0), 150 mM NaCl, 5 mM DTT, 0.1 mg mL^{-1} BSA, 0.03% (v/v) ELUGENT Detergent and 50% (v/v) glycerol. The T7 solution is diluted 50 to 250 times when added to the GUV solution. Those components that cannot go through the α-hemolysin are T7 polymerase and BSA which are both at μM concentration. The T7 concentration is (0.2 U µL^{-1} to 1 U µL^{-1}) which results in 2.4 mM to 12 mM of rNTP incorporation in 12 h at 37 °C. This results in an RNA tile concentration of 6 µM to 35 µM after 12 h of transcription in GUVs. Overall, due to the presence of α-hemolysin, as well as the millimolar concentration of salt and sucrose, the osmolarity of the inner solution fluctuates a negligible amount, preventing osmotic shock. Note that the osmolarity of the outer solution is kept constant against evaporation by sealed chambers and sealed reaction tubes.

5 Supplementary Videos (Videos 1-10)

All confocal time-lapses and z-stacks below contain DiD-labeled GUVs ($\lambda_{ex} = 64$ nm, $\lambda_{em} = 665$ nm) shown in blue and RNA origami (carrying iSpinach-aptamer which binds to the fluorophore DFHBI-1T, $\lambda_{ex} = 488$ nm, $\lambda_{em} = 505$ nm) shown in orange.

Supplementary Video 1: Transcription of 3H-4DT-iSpi RNA origami triggered by rNTPs

This confocal time-lapse captures the expression of 3H-4DT-iSpi RNA origami within GUVs upon rNTPs addition. GUVs contained the DNA template, T7 RNAP and an α-hemolysin pore, which was used to transport the feeding solution (see Methods) along with 6 mM Mg^{2+} except rNTPs for 2 h. The video begins after addition of 4 mM rNTPs and follows RNA origami production for 4 h at 37 °C.

Supplementary Video 2: Transcription of 3H-4DT-iSpi RNA origami triggered by Mg^{2+}

This confocal time-lapse captures the expression of 3H-4DT-iSpi RNA origami within a GUV upon addition of Mg^{2+}. GUVs contained the DNA template, T7 RNAP, the feeding solution (see Methods) along with 4 mM rNTPs, and 1 mM Mg^{2+}. 10 µM Mg^{2+} ionophore was added externally and incubated for 2 h. The video begins after addition of 5 mM Mg^{2+} and follows RNA origami production for 4 h at 37 °C.

Supplementary Video 3: Transcription of S2T RNA origami triggered by rNTPs

This confocal time-lapse captures the expression of S2T RNA origami within GUVs upon addition of rNTPs (see Supplementary Video 1). The video begins after addition of 4 mM rNTPs and follows RNA origami production for 4 h at 37 °C.

Supplementary Video 4: Transcription of iSpi RNA origami tiles triggered by rNTPs

This confocal time-lapse captures the expression of iSpi RNA origami tiles within GUVs upon addition of rNTPs (see Supplementary Video 1). The video begins after addition of 4 mM rNTPs and follows RNA origami production for 4 h at 37 °C.

Supplementary Video 5: Transcription and assembly of RNA origami nanotubes triggered by rNTPs

This confocal time-lapse captures the formation of RNA origami nanotubes within a GUV upon addition of rNTPs (see Supplementary Video 1). The video begins 2 h after addition of rNTPs at 37 °C.

Supplementary Video 6 and 7: Brownian motion of RNA origami nanotubes inside GUVs

These confocal time-lapses capture the fast movement of RNA origami cytoskeletons within GUVs. The videos were recorded after 6 h of transcription triggered by rNTPs addition (see Supplementary Video 1) for 21 sec and 13 sec, respectively. The movement of the RNA cytoskeleton due to Brownian motion is visible.

Supplementary Video 8: RNA origami cortex formation on the inner membrane leaflet of GUVs

This confocal z-stack captures the biotin-aptamer containing RNA origami nanotubes adhering to the biotinylated GUV membrane. RNA origami was expressed inside the GUV for 4 h at 37 °C before the z-stack was taken. The z-projection and 3D construction of this z-stack is shown in Fig. SS24b and Fig. 5d.

Supplementary Video 9: GUV with/without deformation due to RNA nanotube mediated cortex formation

This confocal time-lapse captures a deformed, biotinylated GUV containing RNA nanotubes with biotin aptamer (bottom) and another round, non-deformed biotinylated GUV (top) without RNA nanotube expression in the same microscopic field of view. RNA origami was expressed inside the GUVs for 6 h at 37 °C before the video was taken. The analysis of the deformed GUV is shown in Fig. S25 (second GUV from the top) and Fig. S26 (second GUV from the top left).

Supplementary Video 10: Z-stack of multiple GUVs deformed due to RNA nanotube-mediated cortex formation

These confocal z-stacks capture RNA origami nanotubes with biotin aptamer adhering to the inner surface of the biotinylated GUV membrane. RNA origami was expressed inside the GUVs for 6 h at 37 °C before the z-stack was taken. The analysis of these GUVs is shown in Fig. S25.

References

[1] Geary, C., Grossi, G., McRae, E. K., Rothemund, P. W. & Andersen, E. S. Rna origami design tools enable cotranscriptional folding of kilobase-sized nanoscaffolds. *Nature chemistry* **13**, 549–558 (2021).

[2] Winfree, E., Liu, F., Wenzler, L. A. & Seeman, N. C. Design and self-assembly of two-dimensional dna crystals. *Nature* **394**, 539–544 (1998).

[3] Rothemund, P. W. *et al.* Design and characterization of programmable dna nanotubes. *Journal of the American Chemical Society* **126**, 16344–16352 (2004).

[4] Stewart, J. M., Geary, C. & Franco, E. Design and characterization of rna nanotubes. *ACS nano* **13**, 5214–5221 (2019).

[5] Geary, C. W. & Andersen, E. S. *Design principles for single-stranded rna origami structures*, Vol. 20, 1–19 (International Society for Nanoscale Science, Computation and Engineering, 2014).

[6] Geary, C., Rothemund, P. W. & Andersen, E. S. A single-stranded architecture for cotranscriptional folding of rna nanostructures. *Science* **345**, 799–804 (2014).

[7] O'Neill, P., Rothemund, P. W., Kumar, A. & Fygenson, D. K. Sturdier dna nanotubes via ligation. *Nano letters* **6**, 1379–1383 (2006).

[8] Rivetti, C., Guthold, M. & Bustamante, C. Scanning force microscopy of dna deposited onto mica: Equilibrationversuskinetic trapping studied by statistical polymer chain analysis. *Journal of molecular biology* **264**, 919–932 (1996).

[9] Murugesapillai, D. *et al.* Accurate nanoscale flexibility measurement of dna and dna–protein complexes by atomic force microscopy in liquid. *Nanoscale* **9**, 11327–11337 (2017).
