# Supplementary material for: Genetic encoding and expression of RNA origami cytoskeletons in synthetic cells
Source: Nat Nanotechnol. 2025 Mar 17;20(5):664–71. doi: 10.1038/s41565-025-01879-3 (PMC12095062; doi:10.1038/s41565-025-01879-3)
Supplement: Supplementary file 2 — Reporting Summary [file 41565_2025_1879_MOESM2_ESM.pdf]

nature portfolio

Corresponding author(s): Kerstin Göpfrich

Last updated by author(s): Jan 22, 2025

Reporting Summary

Nature Portfolio wishes to improve the reproducibility of the work that we publish. This form provides structure for consistency and transparency in reporting. For further information on Nature Portfolio policies, see our Editorial Policies and the Editorial Policy Checklist.

Statistics

For all statistical analyses, confirm that the following items are present in the figure legend, table legend, main text, or Methods section.

n/a	Confirmed	
☐	☒	The exact sample size (*n*) for each experimental group/condition, given as a discrete number and unit of measurement
☐	☒	A statement on whether measurements were taken from distinct samples or whether the same sample was measured repeatedly
☐	☒	The statistical test(s) used AND whether they are one- or two-sided<br>*Only common tests should be described solely by name; describe more complex techniques in the Methods section.*
☒	☐	A description of all covariates tested
☒	☐	A description of any assumptions or corrections, such as tests of normality and adjustment for multiple comparisons
☐	☒	A full description of the statistical parameters including central tendency (e.g. means) or other basic estimates (e.g. regression coefficient) AND variation (e.g. standard deviation) or associated estimates of uncertainty (e.g. confidence intervals)
☐	☒	For null hypothesis testing, the test statistic (e.g. *F*, *t*, *r*) with confidence intervals, effect sizes, degrees of freedom and *P* value noted<br>*Give P values as exact values whenever suitable.*
☒	☐	For Bayesian analysis, information on the choice of priors and Markov chain Monte Carlo settings
☒	☐	For hierarchical and complex designs, identification of the appropriate level for tests and full reporting of outcomes
☒	☐	Estimates of effect sizes (e.g. Cohen's *d*, Pearson's *r*), indicating how they were calculated

Our web collection on statistics for biologists contains articles on many of the points above.

Software and code

Policy information about availability of computer code

Data collection	ZEN blue Versoin 3.1 (Carl Zeiss AG), SPMControl Software V7 (Bruker Nano GmbH), Sparkcontrol V 3.2 (Tecan Trading AG)
Data analysis	ImageJ (versions 1.54f & 2.14.0/1.54f), Gwyddion (v2.65), Python (Version 3.10 & 3.11.8, 3.12), matplotlib (v3.9.2), OAT (oxDNA Analysis Tools), GraphPad Prism (Version 5 and 10), ChimeraX (v1.6.1), Adobe Photoshop (v25.12.0); data visualization: Inkscape (versions 1.0.1 & 1.2.2), Adobe Illustrator (v26.5)

For manuscripts utilizing custom algorithms or software that are central to the research but not yet described in published literature, software must be made available to editors and reviewers. We strongly encourage code deposition in a community repository (e.g. GitHub). See the Nature Portfolio guidelines for submitting code & software for further information.

Data

Policy information about availability of data

All manuscripts must include a data availability statement. This statement should provide the following information, where applicable:

- Accession codes, unique identifiers, or web links for publicly available datasets
- A description of any restrictions on data availability
- For clinical datasets or third party data, please ensure that the statement adheres to our policy

The datasets generated during and analysed during the current study are available in the HeidDATA repository on heiDATA /Göpfrich Group - Biophysical Engineering of Life with the identifier https://doi.org/10.11588/data/LJLAMX.

Research involving human participants, their data, or biological material

Policy information about studies with human participants or human data. See also policy information about sex, gender (identity/presentation), and sexual orientation and race, ethnicity and racism.

Reporting on sex and gender	n.a.
Reporting on race, ethnicity, or other socially relevant groupings	n.a.
Population characteristics	n.a.
Recruitment	n.a.
Ethics oversight	n.a.

Note that full information on the approval of the study protocol must also be provided in the manuscript.

Field-specific reporting

Please select the one below that is the best fit for your research. If you are not sure, read the appropriate sections before making your selection.

☒ Life sciences ☐ Behavioural & social sciences ☐ Ecological, evolutionary & environmental sciences

For a reference copy of the document with all sections, see nature.com/documents/nr-reporting-summary-flat.pdf

Life sciences study design

All studies must disclose on these points even when the disclosure is negative.

Sample size	No sample-size calculations were performed. Sample size was determined to be adequate based on the magnitude and consistency of measurable differences between groups.
Data exclusions	Any filters and exclusion applied on the data are described in Statistics and Reproducibility section.
Replication	All experiments were replicated with sufficient technical and experimental replicates determined by the investigators experience with the statistical analysis of common endpoints of our model system as well as the study designs of comparable studies from the literature.
Randomization	GUVs were distributed randomly to all conditions.
Blinding	Investigators were not blinded as it was not compatible with the experimental procedure.

Reporting for specific materials, systems and methods

We require information from authors about some types of materials, experimental systems and methods used in many studies. Here, indicate whether each material, system or method listed is relevant to your study. If you are not sure if a list item applies to your research, read the appropriate section before selecting a response.

Materials & experimental systems

n/a	Involved in the study	
☒	☐	Antibodies
☒	☐	Eukaryotic cell lines
☒	☐	Palaeontology and archaeology
☒	☐	Animals and other organisms
☒	☐	Clinical data
☒	☐	Dual use research of concern
☒	☐	Plants

Methods

n/a	Involved in the study	
☒	☐	ChIP-seq
☒	☐	Flow cytometry
☒	☐	MRI-based neuroimaging

Plants

Seed stocks	n.a.
Novel plant genotypes	n.a.
Authentication	n.a.
